# Cross-platform analysis of cancer microarray data improves gene expression based classification of phenotypes

**DOI:** 10.1186/1471-2105-6-265

**Published:** 2005-11-04

**Authors:** Patrick Warnat, Roland Eils, Benedikt Brors

**Affiliations:** 1 Department of Theoretical Bioinformatics, German Cancer Research Center, Im Neuenheimer Feld 280, D-69120 Heidelberg, Germany; 2 Department of Bioinformatics and Functional Genomics, Institute for Pharmacy and Molecular Biology, University of Heidelberg, Im Neuenheimer Feld 364, D-69120 Heidelberg, Germany

**Keywords:** gene expression profiling, DNA microarray, cross-platform analysis, classification, cancer

## Abstract

**Background:**

The extensive use of DNA microarray technology in the characterization of the cell transcriptome is leading to an ever increasing amount of microarray data from cancer studies. Although similar questions for the same type of cancer are addressed in these different studies, a comparative analysis of their results is hampered by the use of heterogeneous microarray platforms and analysis methods.

**Results:**

In contrast to a meta-analysis approach where results of different studies are combined on an interpretative level, we investigate here how to directly integrate raw microarray data from different studies for the purpose of supervised classification analysis. We use median rank scores and quantile discretization to derive numerically comparable measures of gene expression from different platforms. These transformed data are then used for training of classifiers based on support vector machines. We apply this approach to six publicly available cancer microarray gene expression data sets, which consist of three pairs of studies, each examining the same type of cancer, i.e. breast cancer, prostate cancer or acute myeloid leukemia. For each pair, one study was performed by means of cDNA microarrays and the other by means of oligonucleotide microarrays. In each pair, high classification accuracies (> 85%) were achieved with training and testing on data instances randomly chosen from both data sets in a cross-validation analysis. To exemplify the potential of this cross-platform classification analysis, we use two leukemia microarray data sets to show that important genes with regard to the biology of leukemia are selected in an integrated analysis, which are missed in either single-set analysis.

**Conclusion:**

Cross-platform classification of multiple cancer microarray data sets yields discriminative gene expression signatures that are found and validated on a large number of microarray samples, generated by different laboratories and microarray technologies. Predictive models generated by this approach are better validated than those generated on a single data set, while showing high predictive power and improved generalization performance.

## Background

Gene expression profiling by DNA microarrays has become an important tool for studying the transcriptome of cancer cells, and has been successfully used in many studies of tumour classification and of identification of marker genes associated with cancer [e.g. [1-3]]. With an increasing number of microarray data becoming available, the comparison of studies with similar research goals, e.g. to identify genes being differentially expressed in normal versus tumour tissue, has gained high importance. In general, the evaluation of multiple data sets promises to yield more reliable and more valid results since these results are based on a larger number of samples and the effects of individual study-specific biases are weakened. However, the comparison of results from different microarray studies is hampered by the fact that different studies use different protocols, microarray platforms and analysis techniques. The question whether the results of gene expression measurements obtained by different platforms can be compared has been addressed in several studies [4-7]. It has been found that results derived from the measurements like lists of tumour subtype marker genes [5] or measures of intra-study correlation of gene expression patterns [6] can be compared and thus inter-validated between different platforms. However, the measures of gene expression themselves could not be directly compared between different platforms [4,7]. Some studies propose methods for meta-analysis of microarray data with the goal to identify significantly differentially expressed genes across studies by using statistical techniques that avoid the direct comparison of gene expression values [8-14].

The goal of this study is to investigate the benefit of performing supervised classification analyses across disparate sources of microarray data. Methods of supervised classification analysis render it possible to automatically build classifiers that distinguish among specimens on the basis of predefined class label information (phenotypes), and in many cancer research studies [e.g. [1-3]] the application of these methods has shown promising results of improved tumor diagnosis and prognosis. However, as pointed out by several authors, there is a strong need for independent validation of these results, and an increase in sample size is recommended for future studies [15,16]. We therefore chose to explore how gene expression data from different studies can be directly combined, especially for an integrated classification analysis. Such an integrated analysis promises to be a valuable tool for validation of classification results obtained in a single study, and might yield improved results because it is based on a larger number of samples.

Recently, Wright et al. [17] have proposed a statistical method based on Bayes' rule to classify cancer specimens by their gene expression profiles. They were able to classify oligonucleotide microarray data from one study with a predictor derived from cDNA microarray data from a different study. Here, we evaluate the feasibility of building predictors from and classifying microarray data independent of the platform used for expression profiling. The general approach to first derive numerically comparable measures of gene expression from different platforms (data integration) and then to apply supervised classification on the integrated data was successfully applied in first attempts to classify cancer microarray data generated with multiple array platforms [18,19].

We adopt this approach and demonstrate the use of two data integration methods, namely median rank scores, which has already been successfully applied for comparability assessment of five different breast cancer microarray data sets [19], and quantile discretization which has not been used in the context of microarray data analysis before. For supervised classification analysis, we use support vector machines (SVM), a well-established machine learning technique for classification of microarray data [20,21]. Integrated cross-platform classification of cancer is demonstrated for three pairs of publicly available data from microarray studies on different types of cancer [22-27]. To investigate the hypothesis that an integrated analysis of data from different microarray studies can yield results not obtained by a single study, we chose to investigate two leukemia data sets in more detail and studied differences in gene expression profiles between the cytogenetically defined subgroups t(15;17), t(8;21) and inv(16), all associated with a favourable prognosis [28,29], and samples with normal karyotype lacking mutations in *FLT3 *or *RAS*, thought to belong to an intermediate risk group [30-32]. While differences between the first three groups are prominent and were detected in multiple studies [33,26,27], evidence about the homogeneity of the normal karyotype group and the associated genes is still lacking. The list of genes selected in an integrated analysis of both studies is compared to the lists of genes selected in two analyses performed separately on either study.

## Results

We investigated six publicly available cancer microarray gene expression data sets to perform cross-platform supervised classification analysis. We selected three pairs of studies, each examining the same type of cancer, i.e. breast cancer, prostate cancer and acute myeloid leukaemia, respectively. All pairs of studies allowed for either classification of cancer versus normal tissue or cancer subtype differentiation. Each pair was chosen to consist of one study using cDNA arrays and one study based on oligonucleotide arrays. We studied how to combine pre-processed data sets measured with different microarray platforms for an integrated classification analysis. The process can be divided into the following main parts: First, we determined the overlap of genes common to both platforms using the UniGene database. Next, we derived numerically comparable quantities from the expression values of both platforms by application of median rank scores or quantile discretization. Then, the support vector machine algorithm, an approved method for supervised classification analysis, was applied to different classification settings.

### Data integration

**Figure 1:**
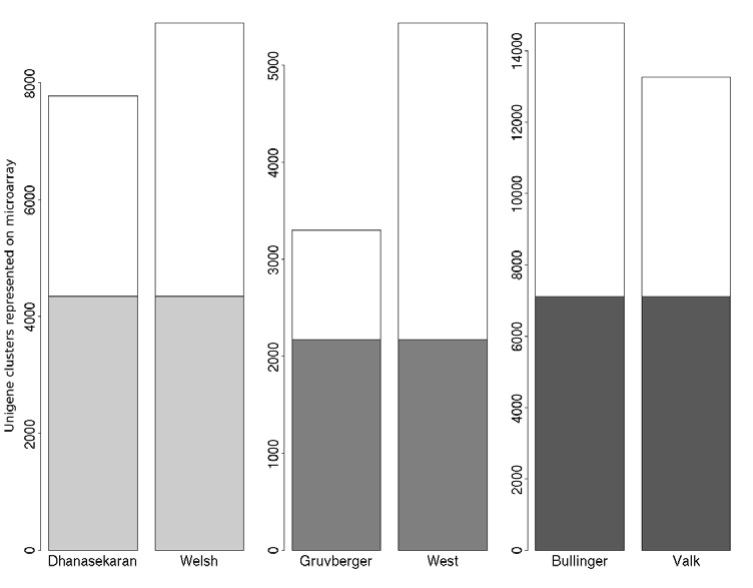
**Barplot of the number of UniGene clusters represented in each data set. **Grey coloured bars indicate the proportion of UniGene clusters common to a pair of studies.

Figure 1 shows for all three study pairs the number of common UniGene clusters (genes) represented on both platforms. Since there is only a moderate overlap of UniGene clusters for the pairs of array platforms, many probes cannot be used for cross-platform analysis. The number of microarray features used for cross-platform analysis is further reduced by averaging expression values of probes on the same platform that map to the same UniGene cluster. As a result, only 40–50% of genes are retained for cross platform analysis.

**Figure 2:**
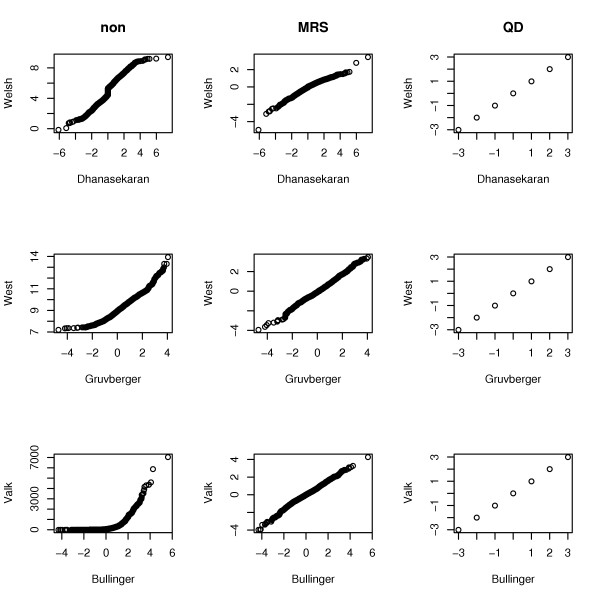
**Quantile-quantile-plots (QQ-plots) comparing the distribution of gene expression values from microarrays of all investigated studies before and after the respective application of MRS or QD. **One microarray per study was selected and a quantile-quantile plot (QQ-plot) for every pair of microarrays from corresponding studies was produced. In every QQ-plot the quantiles of all gene expression values of a first microarray are plotted against the quantiles of all gene expression values of a second microarray. If the gene expression values of the two different microarrays share the same distribution, the points in the plot should form a straight line. Abbreviations: MRS, median rank scores; QD, quantile discretization

As the next step, we applied the median rank scores (MRS) method or quantile discretization (QD). In order to check whether the comparability of the data from different platforms is improved after data transformation by these methods, we compared the distribution of gene expression values per microarray between arrays of different studies. We selected one microarray per study and produced a quantile-quantile plot (QQ-plot) for every pair of microarrays from corresponding studies as shown in Figure 2. In every QQ-plot the quantiles of all gene expression values from a first microarray are plotted against the quantiles of all gene expression values from a second microarray. If the gene expression values of the two different microarrays share the same distribution, the points in the plot should form a straight line. As can be seen in Figure 2, the distribution of expression values of microarrays of different studies is much more similar after application of MRS in comparison to non-integrated data. As an effect of QD, the quantiles of the expression values of all microarrays in the integrated studies are equal by definition, resulting in points in the plots forming a straight line.

### Classification analysis

After data integration by the median rank scores method or quantile discretization, respectively, two different types of cross-platform classification analyses were performed: training of a classifier on only one data set of a pair followed by classifier evaluation on the other data set, and classifier training and testing on data instances randomly chosen by a cross validation from the combined data set.

**Figure 3:**
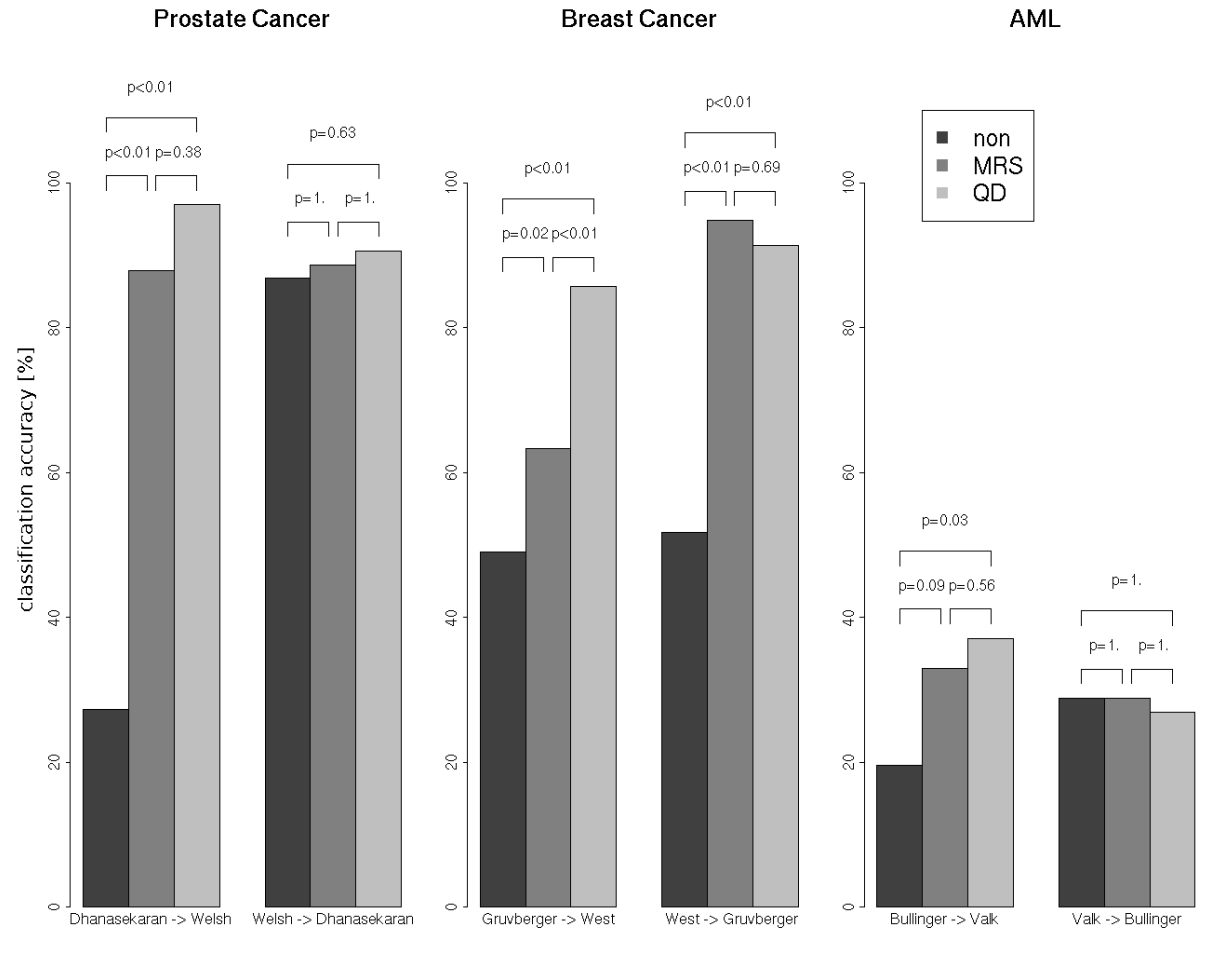
**Barplot of results from a classification analysis using SVM classifiers. **Barplot of results from a classification analysis where all data from one study are used to built a classifier (training), which is then used to classify all samples of the other study (test), using SVM classifiers. The names below the bars indicate which study was used for classifier training (left name) and testing (right name). The bars represent the achieved classification accuracies, i.e. the fraction of samples correctly classified. The colour of a bar indicates the method used for data integration. P-values are obtained by statistical testing with the null hypothesis that the two marked classification approaches perform equally well on the given test set (see Methods for details). The target variable for classification analysis of the prostate cancer data was 'type of tissue' (normal vs. tumor tissue), for the breast cancer data the estrogen receptor (ER) status (ER positive vs. ER negative), and for the leukemia data the karyotype of the samples (one of the chromosomal aberrations t(8;21), t(15;17), inv(16) or normal karyotype, respectively). Abbreviations: MRS, median rank scores; QD, quantile discretization, SVM, support vector machine.

The first type of analysis was performed on non-integrated data and on integrated data, respectively. Evidently, without data integration, a classifier created on one set cannot correctly classify data instances of the other set (Figure 3). This is clearly indicated by prediction accuracies being similar to or worse than the prior prediction rates, i.e. the prediction accuracy of a classifier which always predicts a data instance to be an element of the dominating class. The only exception is the prostate cancer data, where high classification accuracy was achieved after training on the data set of Welsh et al. and classification of the data of Dhanasekaran et al. Data integration improves the results in cases of the prostate and breast cancer studies (p-values < 0.01, except for classification of the data of Dhanasekaran et al, where a high classification accuracy was already achieved on the non-integrated data set). We conclude for these two pairs of studies that data integration enables the successful application of classifiers trained on one data set to a comparable data set generated with a different platform. This conclusion does not hold for the AML studies. Here, only the result for building a classifier based on the data of Bullinger et al. and classifying the data of Valk et al. improved after application of median rank scores or quantile discretization (p-value < 0.1).

Except for the pair of breast cancer microarray data sets, the application of the MRS versus QD showed no significantly different effect on the achieved classification accuracies. For the training on the data set of Gruvberger et al. and classification of the data of West et al., the classification result was significantly better after application of QD in comparison to the result obtained after using the MRS method. In all other cases both methods can be considered equivalent.

In addition to the above mentioned separated training and validation, cross-validation analyses were performed on combined data sets. High classification accuracies were achieved with training and testing on data instances randomly chosen from both data sets (> 85%; see table 2). Although the integrated classifiers only operated at less than 50% of all genes, classification accuracies for integrated classifiers were nearly as high or even markedly improved in comparison with classification accuracies achieved for single data sets only. In the case of the breast cancer studies, the results were better than the accuracies achieved by cross-validation on each of the pre-processed single sets with all available microarray features.

**Table 2:** Classification results observed by cross validation using SVM classifiers. Figures represent achieved classification accuracies, i.e. the fraction of samples correctly classified. The upper table shows results for cross validation analysis of both data sets of a pair, where samples for training and testing are selected randomly from both studies. For this, data sets were integrated by either MRS or QD. The bottom table contains the results of a cross-validated classification analysis performed separately on each study, using all available gene expression data after pre-processing (without applying MRS or QD). Abbreviations: MRS, median rank scores; QD, quantile discretization, SVM, support vector machine.

***both data sets integrated***		
	MRS	QD
Prostate cancer	97.67 %	97.56 %
Breast cancer	87.01 %	88.97 %
Acute myeloid leukemia	90.60 %	90.20%
		
***original data***		
Prostate cancer	Dhanasekaran et al.	Welsh et al.
	95.28 %	99.09 %
Breast cancer	Gruvberger et al.	West et al.
	80.52 %	86.73 %
Acute myeloid leukemia	Bullinger et al.	Valk et al.
	68.53 %	99.90 %

In order to check whether similar classification results could be obtained with another method of supervised classification analysis, we repeated the above described experiments using the method of nearest shrunken centroids classification (also known as "Prediction Analysis of Microarrays", PAM) [34]. As presented in Additional Files 1 and 2, the classification results obtained with PAM are similar to those obtained by SVM.

### Selection of genes with discriminative expression patterns

**Figure 4:**
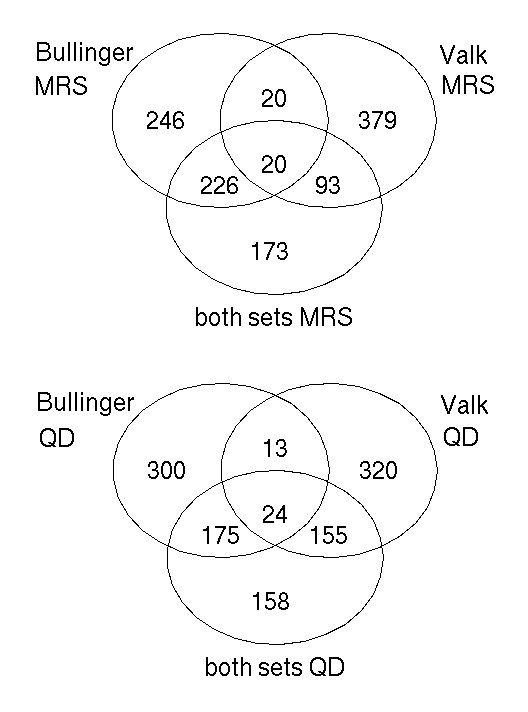
**Venn diagrams showing the overlap between lists of genes generated by RFE analysis. **Venn diagrams showing the overlap between lists of genes generated by RFE analysis based on single sets (Bullinger et al. or Valk et al.) and based on both data sets integrated by MRS or QD. Abbreviations: MRS, median rank scores; QD, quantile discretization, RFE, recursive feature elimination.

To show the potential of an integrated cross platform analysis, we generated lists of genes forming discriminative expression patterns by means of recursive feature elimination (RFE) analysis for the leukemia studies (see Methods for details). We generated six lists of genes, two lists for an analysis of the combined leukemia studies, integrated by MRS or QD, and two lists for each of the two leukemia studies analysed separately, using only samples of the either the MRS or QD data which belong to one study. A number of 512 elements was selected for each list, which corresponded to minimal cross-validated error rate in the integrated analyses of data from both leukemia studies. Interestingly, the intersection of the lists generated in analyses using only data of one of the two leukemia studies comprises only about 40 UniGene clusters, independently of whether MRS or QD was used (Figure 4). In the sets generated by an analysis of both studies together, integrated by MRS or QD, many genes were selected that were lost in the analyses based on a single study (Figure 4). These include important genes with regard to the biology of leukemia, like *RXRA*, *PBX3*, *ABL2*, *SOCS1*, and *EGR2 *(see Additional File 3 for annotated lists of selected genes; Additional File 4 contains all six gene lists ordered by gene ranks as determined in RFE analysis).

**Figure 5:**
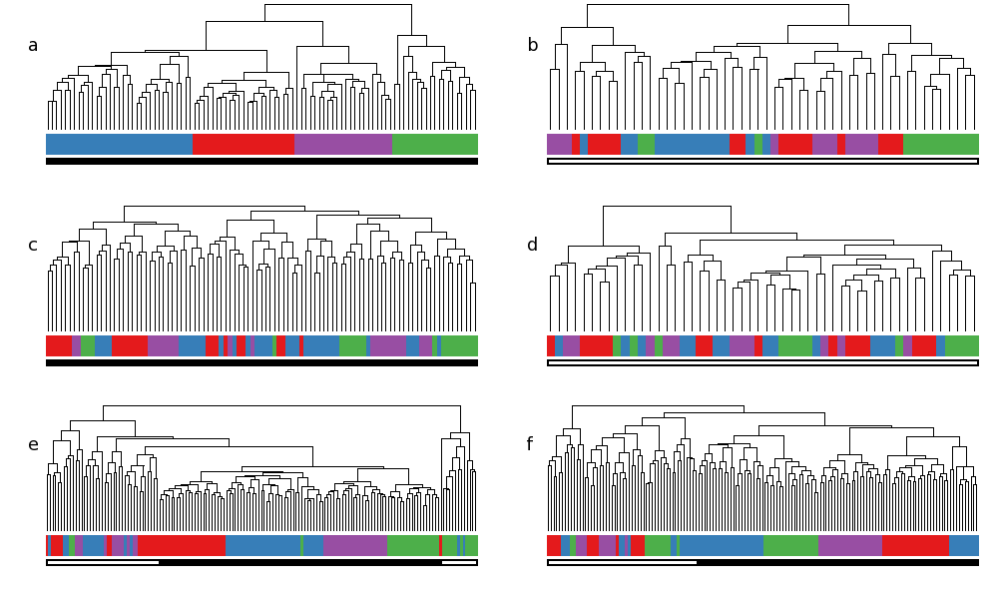
**Hierarchical clustering of leukemia samples. **Hierarchical clustering of leukemia samples based on expression values of genes selected by RFE analysis. The colored bars indicate the true class affiliations of every sample, the black and white bars below indicate study origin. (a) Clustering result for data from Valk et al. or (b) Bullinger et al. using only genes selected by RFE on this data set. (c) Clustering of data from Valk et al. after data integration by MRS algorithm using only expression values of genes selected by RFE on the data of Bullinger et al. (d) Clustering of data from Bullinger et al. based on genes selected on data from Valk et al. Data integrated by QD or non-integrated data yielded results similar to those here (data not shown). (e) Clustering results of all samples of both studies using gene lists generated on the combined set integrated by MRS or (f) QD. Abbreviations: MRS, median rank scores; QD, quantile discretization, RFE, recursive feature elimination.

Finally, we used hierarchical clustering as a visualization method to display coherence in gene expression of the genes selected by RFE in the leukemia studies. We first clustered the data of both leukemia studies separately based on the genes selected by RFE on either set. As shown in Figure 5(a,b), the samples of Valk et al. were perfectly grouped according to their karyotype while in the data of Bullinger et al. samples with karyotype t(8, 21) and inv(16) were not grouped homogeneously. Then, we clustered the data of Valk et al. using only genes found to be discriminative on the data of Bullinger et al. (Figure 5c). Figure 5d shows the reverse case, a clustering of the data of Bullinger et al. based on the gene selection on the data of Valk et al. For the selected groups of genes, coherence in gene expression between samples of the same karyotype was weak when results of an analysis solely based on one leukemia data set are transferred to the other leukemia data set, as samples of the same karyotype were not grouped homogeneously. Figure 5e and 5f show clustering results on all samples of both studies using gene lists integrated either by MRS or QD. Here we can observe a much more consistent grouping of the samples according to their karyotype than that observed in Figure 5c and 5d. Still, both methods of data integration are not able to fully eliminate study specific self-similarity of samples, as the samples form clusters according to study origin.

## Discussion

In this study we showed that classification of cancer microarray data can be markedly improved by cross-platform classification analysis of gene expression data from different studies with similar focus. Key techniques for cross-platform classification analysis were data integration methods rendering microarray data numerically comparable across platforms in combination with well-established machine learning techniques for generation of predictive models. An obvious advantage of an integrated classification analysis is the improved generalization performance and reliability of the resulting predictive models (classifiers) since they are found and validated on a larger number of samples, thus the effect of study-specific biases can be reduced. For all study pairs used here, we achieved high classification accuracies when using data samples randomly chosen from both data sets of a comparison pair for classifier building and testing. Our findings endorse the encouraging results of first attempts of multi-platform microarray classification analysis [18,19].

For integration of microarray measurements from different platforms, Bloom et al. [18] used a scaling approach based on measurements for one common reference RNA sample. As hybridization results for such a common reference RNA sample are normally not available for different microarray studies and platforms (especially in the case of custom made cDNA arrays), we applied the median rank scores method [19] and quantile discretization for data integration. Besides the problem of integrating microarray data that have been measured with different platforms, a general problem in combining measurements from different gene expression studies is the variability between results of different studies. This is primarily due to biological differences among the samples of different studies, differences in the technical procedures to obtain gene expression measurements, and random variation. The use of methods providing an abstraction of data like ranks or discretized values reduces this variability at the price of reduced information. Therefore, data sets processed by MRS or QD can not be considered as a suitable input for every kind of analysis purpose. However, for the aim of cross-platform classification analysis, the combination of such abstraction methods with a sophisticated machine learning technique like the support vector machine used here helps to compensate for this loss of precision, and can yield useful results.

Even when a classifier is built on one data set of a pair of compared studies and the samples of the other study are classified, good classification results can be observed for the prostate and breast cancer studies. In this case, the generalization ability of the classifier is sufficient to correctly classify most of the samples of the other study, and thus the classifier obtained on the data of one study can be validated by the data of another study. In contrast, the results for the AML studies indicate that the generated classifiers based on only one of these studies are too specific. This might be due to fact that the sample sets of either study are not representative enough to cover all characteristic transcriptional features observable for the investigated phenotypes. Indeed, the results for the cross-validation analysis using samples from both AML studies show that classifiers with better generalization performance can be obtained underlining the potential of a cross-platform classification analysis.

Selection of discriminative gene expression signatures is an important task frequently performed in microarray studies. Here, we applied RFE analysis for selecting subsets of genes with distinctive expression patterns on the data of the leukemia studies of Bullinger et al. [26] and Valk et al. [27]. For visualization of the coherence in gene expression of the genes selected by RFE in the different studies we performed hierarchical clustering. Gene sets selected only on data of one study show poor coherence in gene expression for the karyotype groups of samples on the other set. Clustering results observed for gene sets selected on the combined set are more consistent. Therefore, these discriminative gene sets are apparently of more general validity. On the other hand, cluster analysis showed that neither of the two methods of data integration was able to entirely overcome study specific self-similarity of the leukemia samples. For cross-platform classification analysis, however, the MRS and QD algorithms yielded good results.

The analysis of gene lists obtained by RFE indicated that gene signatures can be generated on a combined set that comprise important genes that were not part of gene signatures generated on either set alone. Notably, the intersection of lists from the Bullinger and Valk data sets with the list from the combined set contained only a few genes, none of them to be known of high importance in the context of AML. Similarly, the intersection between the Bullinger and Valk data sets was not large (Additional File 3). In contrast, the list obtained from the combined data set contained many genes well known to be involved in leukemia pathogenesis, like *PBX3 *[35], the retinoid receptor *X *[36], the *ABL2 *tyrosine kinase [37] or early growth response 2 [38]. In addition, many genes in the combined list are prominent oncogenes or tumour suppressors, like *BCL2 *[39] or *ERBB3 *[40]. Most notable is the inclusion of human telomerase *TERT*, which has been found by Hahn et al. to be one of three necessary factors for transforming a normal cell into a tumour cell [41].

We compared the gene lists generated by the RFE method to the result of a meta-analysis approach as described by Rhodes et al. [8]. This method aims at identifying genes that show reproducible standardized differences in mean expression between phenotype groups across studies. For this, a p-value is calculated for every gene in both leukemia studies separately, in order to quantify the significance of differences in mean expression between phenotype groups within a study. Then, the study specific p-values are combined to a test statistic *S *and significance values for this test statistic by a permutation approach are calculated (for details see [8]). At a significance level of p = 0.01, 43 genes were selected by this meta-analysis approach. Of these 43 genes, 12 genes were also found in the list generated by an RFE analysis of the data of both studies integrated by MRS, 19 were also found in the list generated by an RFE analysis of the data of both studies integrated by QD. This result shows that the gene lists selected by RFE analysis also contains genes that would have been found in an independent meta-analysis, but that also many different genes are selected. This is not surprising, as there are essential differences in both approaches. The meta-analysis performed here applies a univariate statistical test to find genes with a significant difference in group means of expression values, whereas the SVM based RFE analysis is a multivariate approach which also considers combinations of genes and selects genes with maximum influence on the discriminative performance of a classifier. While interpreting a gene list generated in a RFE analysis, one has to keep in mind that the main goal of methods like the SVM based RFE approach used here is to generate signatures that allow for accurate classification of phenotypes. These gene signatures are unlikely to contain all and only genes that are most relevant to the genetic differentiation between complex disease phenotypes. The task to find the complete set of only those relevant genes out of gene expression data is much more demanding and might pose an irresolvable challenge as the changes of gene expression profiles recorded by microarrays are mostly secondary and tertiary effects and not the primary ones. With microarrays one observes the avalanche of gene expression changes, not necessarily the small pebble causing it. First promising concepts and methods to work on the task to find the set of relevant genes have been proposed [42], but their usefulness to address biological questions has still to be thoroughly investigated. However, the finding that RFE signatures generated by an integrated analysis of both leukemia studies contained genes that are described as being relevant for tumor biology in the literature, and that were not found in either single set analyses, shows the potential of cross-platform microarray data integration to be useful not only to improve results for phenotype classification but also for generation of gene signatures that contain more biologically interesting genes.

Considering integrated classification analyses in general, a limiting factor for future application is posed by inconsistencies in biological phenotype annotation across studies. In many cases, it is hard to obtain consistent annotation on the samples used that would allow to form comparable groups for classification analysis. This is due to lack of ontologies for description, or the use of categories that are based on subjective evaluation such as histological grading or borderline expression of a molecular marker as determined by immunohistochemistry. In such respect, it would be highly desirable to introduce systems for annotation of samples that are analogous to the MIAME standard for description of technical details of hybridization [43]. Until such a system exists, one has to focus on studies where consistency can be guaranteed by expert evaluation, as is the case for the data sets investigated here.

More study results will be needed to validate our findings. Cross-platform analyses have to be conducted considering more than two studies at a time. Here, the problem of having relatively few genes in common between all studies will gain increasing importance. Methods to make use of gene expression values only available on some platform(s) but not on others will be required. For this, the adaption of a recent approach by Guo et al. [44] could be a first step. Guo et al. use functional expression profiles (FEP) instead of gene expression profiles (GEP) for their classification analysis and generate the FEP by averaging the expression levels of genes mapping to the same Gene Ontology (GO) annotation. For integrating data from different microarray platforms, mapping of such functional summary measurements as FEP rather than the actual gene expression measurements between different chip platforms might result in an increased number of measurements (in terms of the number of genes) having an influence on the analysis results. However, by this approach the amount of information in the data is also reduced, as for example anti-correlated genes mapping to the same GO annotation would countervail each other. Further research is required to evaluate the impact of these two effects on the results of an integrated cross-platform classification analysis. The general improvement of matching genes between different platforms would also be beneficial in order to avoid false or missing mappings. Such developments are under way in our laboratory.

## Conclusion

Cross-platform classification of multiple cancer microarray data sets yields discriminative gene expression signatures that are found and validated on a large number of microarray samples, generated by different laboratories and microarray technologies. Predictive models generated by this approach are better validated than those generated on a single data set, while showing high predictive power and improved generalization performance. The results presented here for the three sample study pairs indicate that this approach bears the potential to become a widely applicable technique for inter-validation of studies performing classification of microarray gene expression data.

## Methods

### Gene expression data collection and preprocessing

All data for this study were downloaded from public web sites (Table 1) and were pre-processed by software packages included in the R-project [45] or Bioconductor [46], respectively. For all studies where raw microarray data were available, pre-processing was performed as follows. Microarray features with more than 20% missing values across all arrays per study were not considered for further analysis. Missing values for all remaining features were replaced by median values per gene. Normalization was carried out using either the vsn [47] or loess [48] algorithms with default parameters as implemented in the Bioconductor software packages vsn and marray. Data were base-two log-transformed where applicable.

**Table 1:** Key characteristics of used microarray data. The figures in curly brackets denote the number of samples belonging to each category. The number of probes comprises all probes for which data were available and which have not been filtered out in the preprocessing of the data (see methods for details). Abbreviations: ER, estrogen receptor status; AML, acute myeloid leukemia; t(A;B), balanced translocation of genetic material between chromosomes A and B; inv(16), inversion of a segment of chromosome 16; NN, normal karyotype.

***Study***	***Cancer***	***Platform***	***Samples***	***Probes***	***Target Variable of Classification Analysis***
Dhanasekaran et al[22]	Prostate cancer	cDNA	53	7769	Tissue: tumor{34}, normal{19}
Welsh et al[23]	Prostate cancer	oligo	33	9023	Tissue: tumor{24}, normal{9}
Gruvberger et al[24]	Breast cancer	cDNA	58	3300	ER-status: positive{28}, negative{30}
West et al[25]	Breast cancer	oligo	49	5435	ER-status: positive{25}, negative{24}
Bullinger et al[26]	AML	cDNA	52	14776	Karyotype: t(8;21){11},t(15;17){12},inv(16){15}, NN{14}
Valk et al[27]	AML	oligo	97	13250	Karyotype: t(8;21){22}, t(15;17){19},inv(16){23}, NN{33}

### Data integration

The UniGene database (Build 171) [49] was used to match cDNA clones and Affymetrix probe sets between platforms. Each transcript from the different microarrays was mapped to a UniGene cluster. The overlap of genes was determined by forming the intersection of the respective UniGene cluster sets. Within each study, expression values corresponding to probes of the same UniGene cluster were averaged. Genes that did not map to any UniGene Cluster and genes not mapping to a UniGene cluster obtained for the other microarray platform were not considered for cross-platform analysis.

In the case of the breast cancer data sets [24,25], all probes corresponding to the estrogen receptor gene (UniGene cluster Hs.1657) have been removed for further analysis since, for these data sets, the estrogen receptor status of the samples should be predicted independently of the expression of the estrogen receptor gene.

For the comparison of the leukemia microarray data sets [26,27], we selected only those samples belonging to one of the following karyotypes being represented in both data sets: t(8;21), t(15;17), inv(16) and normal karyotype, respectively.

To derive numerically comparable measures of gene expression for different microarray platforms we used either median rank scores or quantile discretization. Before either of these methods was applied to the preprocessed data, all expression values of oligonucleotide arrays were divided by the median expression value per array to scale absolute intensity values to relative ratio values.

#### Median Rank Scores

(MRS) [19] The basic idea of this method is to transform gene expression values of different microarray platforms to a common numerical range by replacing numerical values of one study by numerical values from the other study, with respect to the relative ranks of expression values within each study. Therefore, one of the microarray data sets to be compared is chosen as a reference set. For each gene, the median expression value over all microarrays of the reference study is calculated, and the resulting vector of median gene expression values is sorted in ascending order. Next, for every microarray of the non-reference set, the relative rank of each gene expression value is determined. An expression value with rank *n *is then replaced by element *n *of the sorted median expression vector. Thus, the gene expression values of all microarrays of the non-reference sets are replaced by surrogate values with comparable numerical range relative to the reference data set. Therefore, the study comprising most microarrays should be designated as the reference set. Under certain circumstances it might make sense to chose the reference set according to another criterion than sample size, e. g. when the largest data set shows an inferior expression data quality in comparison to the smaller sets. Note that the only information being preserved for the non-reference set are the relative ranks of gene expression values. To keep our analyses comparable with regard to the selection of the reference set, we always selected the study using a cDNA microarray as reference data set because for two of the three investigated pairs of studies the study using a cDNA microarray contained more samples (microarrays) than the corresponding study that used an oligonucleotide microarray.

#### Quantile discretization

(QD) This method is based on equal frequency binning [50]. Here, the expression values of all arrays are discretized into a predetermined number of bins *b (b = 8) *for all our analyses. For each experiment, *b *subsets with equal number of values are determined using the quantiles of the array expression values as cut points, where a cut point is here defined as the expression value separating an ordered set of expression values into two subsets. The two central bins with the median value as cut point are merged into one bin yielding one central interval. Every expression value is replaced by an integer value corresponding to the bin it falls into, where zero is assigned to central bin and all other bins are numbered consecutively beginning with the bins next to the central one, using positive integers for bins containing values above the median and negative integer values for the others. Both methods were implemented using the R software for statistical computing [45]. Code is available upon request.

### Classification analysis

For each pair of studies, classification analyses were performed on the UniGene matched gene expression values. We investigated how well a classifier trained on one data set predicts class labels of the other data set after application of MRS and QD, respectively, compared to no application of MRS or QD. For each pairwise combination of these three approaches, a statistical test with the null hypothesis of equal performance in classification of the given test set was realized according to Salzberg [51]: For comparing the performance of two classification approaches A and B on a given test set, the number of test samples *n *for which one of the two approaches gave a correct classification and the the other approach gave a wrong classification is determined. If both approaches perform equally well, then among these *n *samples the proportion *p *of samples for which approach A gave a correct classification should be equal to the proportion *q *of samples for which approach B gave a correct classification. Therefore, the null hypothesis of equal classification performance of A and B can be tested by a binomial test with null hypothesis *p = q = 0.5*.

In addition, we examined the class prediction accuracies by 10-times repeated (i.e. 10 resampling replicates) 10-fold cross-validation. Arrays of both studies were chosen randomly for training and testing after data integration by the median rank scores method and by quantile discretization, respectively. Finally, we performed a cross-validated classification analysis on each data set alone using all available pre-processed gene expression values.

We used support vector machines (SVM) for supervised classification analysis, applying the libsvm implementation by Chang and Lin with a polynomial kernel function [52]. Hyperparameters *C *and *degree *were tuned by cross-validating parameter combinations in a grid search over a two-dimensional parameter space with ranges from 2^-5 ^to 2^10 ^and 1 to 3, respectively.

For classification with nearest shrunken centroids (PAM), we used the corresponding R package pamr, available on the Bioconductor website [46]. The hyperparameter *delta *(threshold for centroid shrinkage) was tuned over the default parameter range given in the pamr package.

Parameter tuning for both classification methods was done by a three-fold cross-validation and was repeated for cross-validation in each single iteration (nested cross-validation). No variable pre-selection was performed on the preprocessed data prior to classifier construction. The scheme of our workflow for calculating class prediction accuracies is shown in Additional File 5.

**Figure 6:**
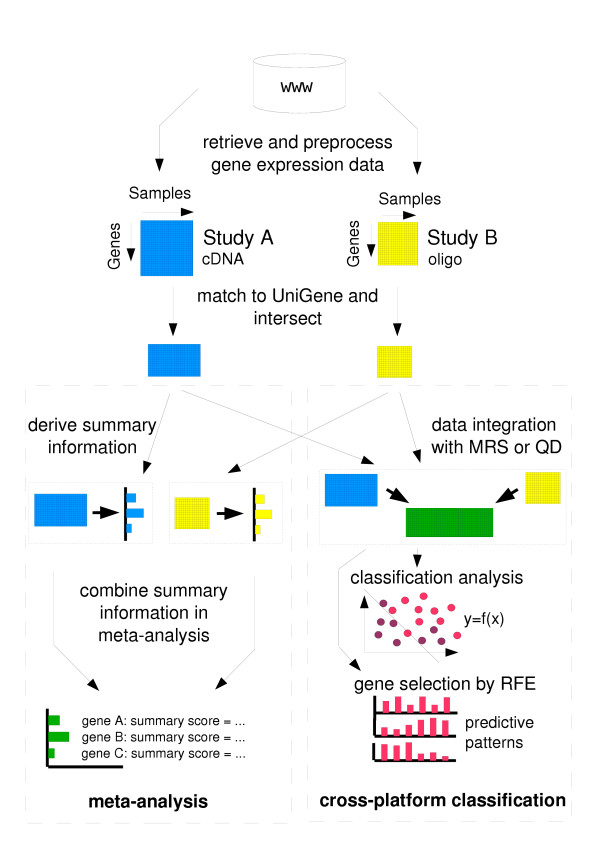
**Flow diagram of the presented cross-platform classification approach. **Flow diagram of the presented cross-platform classification approach (see Methods for details) compared to a meta-analysis approach.

The whole process of cross-platform classification analysis in comparison to a meta-analysis approach is summarized in Figure 6.

### Selection of genes with discriminative expression patterns

Independently of the classification analysis described above, we applied a SVM based Recursive Feature Elimination (RFE) method [53] for selection of genes with discriminative expression patterns in case of the leukemia studies by Bullinger et al. (2004) and Valk et al. (2004). We used an implementation of the method in R [54]. As the magnitude of the internal SVM classifier feature weights represent the influence of a feature on a classification decision by that classifier, the approach suggested by Guyon et al. [53] uses the internal feature weights of an SVM classifier to generate a feature ranking. This is realised by repeatedly fitting an SVM model to given data and iteratively eliminating features from this model. We generated six lists of genes, two lists for an analysis of both leukemia studies together, integrated by MRS or QD, and two lists for each of the two leukemia studies analysed separately, using only samples of either the MRS or QD data which belong to one study. Note that in the integrated analyses as well as in the analyses based on single study data only expression data for only those genes were used that were present on both microarray platforms used in the two studies. For generation of gene lists with RFE, we first performed a 10-fold cross-validation once on every given data set for optimizing the number of selected genes, where we only considered gene lists containing a number of genes equal to a power of two but less than the total number of genes. For the two integrated analyses of data from both leukemia studies, a number of 512 elements corresponded to the minimal cross-validated error rate. We next applied RFE to every dataset (without cross-validation) resulting in one ranking of all genes per data set. We then selected the 512 most highly ranked genes for every data set and finally compared the six different lists of 512 genes.

Moreover, we visualized results from RFE analysis by performing hierarchical clustering of the leukemia data based on the generated gene lists. For hierarchical clustering, we used the method "hclust" of the R package mva, applying the following parameter settings: Manhattan distance function was performed on data transformed to zero mean and unit variance, and clustering was done using a complete linkage algorithm [55].

## List of abbreviations

AML: acute myeloid leukemia

FEP: functional expression profile

GEP: gene expression profile

GO: gene ontology

MIAME: minimum information about a microarray experiment

MRS: median rank scores

PAM: prediction analysis of microarrays

QD: quantile discretization

QQ-plot: quantile-quantile plot

RFE: recursive feature elimination

SVM: support vector machine

## Authors' contributions

PW conceived of the study, carried out the analyses and drafted the manuscript. RE participated in the design of the study and helped to draft the manuscript. BB coordinated the study, participated in its design, performed the hierarchical cluster analysis and helped to draft the manuscript. All authors read and approved the final manuscript.

## Supplementary Material

Additional File 1 Barplot of results from a classification analysis where all data of one study is used to built a classifier (training), which is then used to classify all samples of the other study (test), using PAM classifiers. The names below the bars indicate which study was used for classifier training (left name) and testing (right name). The bars represent the achieved classification accuracies, i.e. the fraction of samples correctly classified. The colour of a bar indicates the method used for data integration. P-values are obtained by a statistical test with the null hypothesis that the two marked classification approaches perform equally well on the given test set (see methods for details). The target variable for classification analysis of the prostate cancer data was 'tissue type' (normal vs. tumor tissue), for the breast cancer data the estrogen receptor (ER) status (ER positive vs. ER negative), and for the leukemia data the karyotype of the samples (one of the chromosomal aberrations t(8;21), t(15;17), inv(16) or normal karyotype, respectively). Abbreviations: MRS, median rank scores; QD, quantile discretization, PAM, prediction analysis of microarrays. Click here for file

Additional File 2 Classification results observed by cross validation using PAM classifiers. Figures represent achieved classification accuracies, i.e. the fraction of samples correctly classified. The upper table shows results for cross validation analysis of both data sets of a pair, where samples for training and testing are selected randomly from both studies. For this, data sets were integrated by either MRS or QD. The bottom table contains the results of a cross-validated classification analysis performed separately for each study, using all available gene expression data after pre-processing (without application of MRS or QD). Abbreviations: MRS, median rank scores; QD, quantile discretization, PAM, prediction analysis of microarrays. Click here for file

Additional File 3 The overlap between lists of genes found by RFE analysis based on single sets (Bullinger et al. or Valk et al.) and based on both data sets integrated by MRS or QD. Abbreviations: MRS, median rank scores; QD, quantile discretization, RFE, recursive feature elimination. Click here for file

Additional File 4 All six lists of genes found by RFE analysis (see Methods for details). In every list, the corresponding UniGene identifiers of the genes are ordered according to their rank as determined in the RFE analysis. Abbreviations: RFE, recursive feature elimination. Click here for file

Additional File 5 Workflow for calculation of the presented class prediction accuracies. (a) Classifier performance evaluation on an independent data set as applied for calculation of the results presented in Figure 3 and Additional File 1. (b) Classifier performance evaluation by repeated cross validation as applied for calculation of the results presented in Table 2 and Additional File 2. Click here for file
