# Supplementary figures and images for: Cross-platform analysis of cancer microarray data improves gene expression based classification of phenotypes
Source: BMC Bioinformatics. 2005 Nov 4;6:265. doi: 10.1186/1471-2105-6-265 (PMC1312314; doi:10.1186/1471-2105-6-265)

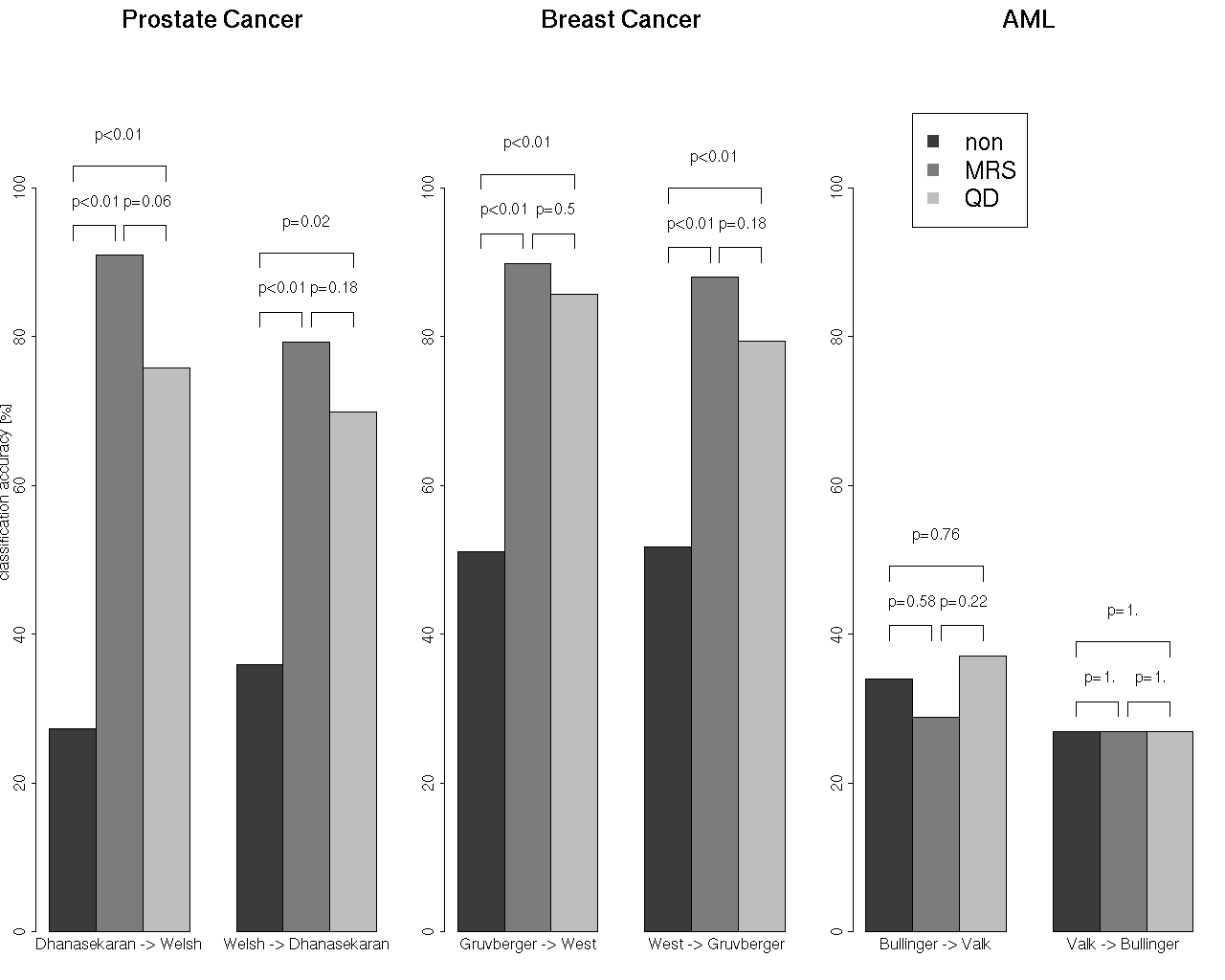

Supplement: Additional File 1 — Barplot of results from a classification analysis where all data of one study is used to built a classifier (training), which is then used to classify all samples of the other study (test), using PAM classifiers. The names below the bars indicate which study was used for classifier training (left name) and testing (right name). The bars represent the achieved classification accuracies, i.e. the fraction of samples correctly classified. The colour of a bar indicates the method used for data integration. P-values are obtained by a statistical test with the null hypothesis that the two marked classification approaches perform equally well on the given test set (see methods for details). The target variable for classification analysis of the prostate cancer data was 'tissue type' (normal vs. tumor tissue), for the breast cancer data the estrogen receptor (ER) status (ER positive vs. ER negative), and for the leukemia data the karyotype of the samples (one of the chromosomal aberrations t(8;21), t(15;17), inv(16) or normal karyotype, respectively). Abbreviations: MRS, median rank scores; QD, quantile discretization, PAM, prediction analysis of microarrays. [file 1471-2105-6-265-S1.png]
