# Supplementary material for: Cross-platform analysis of cancer microarray data improves gene expression based classification of phenotypes
Source: BMC Bioinformatics. 2005 Nov 4;6:265. doi: 10.1186/1471-2105-6-265 (PMC1312314; doi:10.1186/1471-2105-6-265)
Supplement: Additional File 2 — Classification results observed by cross validation using PAM classifiers. Figures represent achieved classification accuracies, i.e. the fraction of samples correctly classified. The upper table shows results for cross validation analysis of both data sets of a pair, where samples for training and testing are selected randomly from both studies. For this, data sets were integrated by either MRS or QD. The bottom table contains the results of a cross-validated classification analysis performed separately for each study, using all available gene expression data after pre-processing (without application of MRS or QD). Abbreviations: MRS, median rank scores; QD, quantile discretization, PAM, prediction analysis of microarrays. [file 1471-2105-6-265-S2.pdf]

## Additional file 2 - Classification results observed by cross validation using PAM classifiers

| | | |
|---|---|---|
| ***both data sets integrated*** | | |
| | MRS | QD |
| Prostate cancer | 95.35 % | 97.67 % |
| Breast cancer | 87.85 % | 92.52 % |
| Acute myeloid leukemia | 71.14 % | 68.46% |
| | | |
| ***original data*** | | |
| Prostate cancer | Dhanasekaran et al. | Welsh et al. |
| | 92.45 % | 93.94 % |
| Breast cancer | Gruvberger et al. | West et al. |
| | 94.83 % | 83.67 % |
| Acute myeloid leukemia | Bullinger et al. | Valk et al. |
| | 76.92 % | 98.97 % |
