# Supplementary material for: Cross-platform analysis of cancer microarray data improves gene expression based classification of phenotypes
Source: BMC Bioinformatics. 2005 Nov 4;6:265. doi: 10.1186/1471-2105-6-265 (PMC1312314; doi:10.1186/1471-2105-6-265)
Supplement: Additional File 3 — The overlap between lists of genes found by RFE analysis based on single sets (Bullinger et al. or Valk et al.) and based on both data sets integrated by MRS or QD. Abbreviations: MRS, median rank scores; QD, quantile discretization, RFE, recursive feature elimination. [file 1471-2105-6-265-S3.pdf]

MRS intersect of all sets

UnigeneID	Description	Symbol	Cytoband	LocusLinkID	Acc No
Hs.26985	disrupted in schizophrenia 1	DISC1	1q42.1	27185	AJ506177.1;
Hs.301011	jumonji domain containing 2B	JMJD2B	19p13.3	23030	BC040014.1;
Hs.76536	transducin (beta)-like 1X-linked	TBL1X	Xp22.3	6907	Y12781.1;
Hs.50216	zinc finger protein (ZFD25)	ZFD25	7q11.2	51427	AB014735.1;
Hs.211588	POU domain, class 4, transcription factor 1	POU4F1	13q21.1-q22	5457	BC041859.1;
Hs.3407	protein kinase (cAMP-dependent, catalytic) inhibitor gamma	PKIG	20q12-q13.1	11142	AF182032.1;
Hs.278429	chromosome 9 open reading frame 78	C9orf78	9q34.2	51759	AF007871.1;
Hs.90063	neurocalcin delta	NCALD	8q22-q23	83988	BC036098.1;
Hs.8309	likely ortholog of mouse membrane bound C2 domain containing protein	MBC2	12q13.13	23344	BC013880.2;
Hs.15871	lysophosphatidic acid phosphatase	ACP6	1q21	51205	BC034686.1;
Hs.108806	AT-binding transcription factor 1	ATBF1	16q22.3-q23.1	463	L32833.1;
Hs.421474	ATP-binding cassette, sub-family A (ABC1), member 5	ABCA5	17q24.3	23461	NM_172232.1;
Hs.14158	copine III	CPNE3	8q21.2	8895	BC036242.1;
Hs.16134	serine/threonine kinase 10	STK10	5q35.1	6793	AK092814.1;
Hs.153563	lymphocyte antigen 75	LY75	2q24	4065	NM_002349.1;
Hs.436348	potassium channel tetramerisation domain containing 15	KCTD15	19q13.12	79047	AK096843.1;
Hs.371698	RAE1 RNA export 1 homolog (S. pombe)	RAE1	20q13.31	8480	U84720.1;
Hs.226117	H1 histone family, member 0	H1F0	22q13.1	3005	AK091372.1;
Hs.416848	cathepsin W (lymphopain)	CTSW	11q13.1	1521	AF013611.1;
Hs.224397	transcription elongation factor A (SII), 2	TCEA2	20q13.33	6919	BC018896.2;

Table A: The list of genes found by RFE in all MRS sets.

This list is the intersection of the three gene lists generated on the data of Bullinger et al, Valk et al and of the list generated on the data of both studies integrated by MRS. Abbreviations: MRS, median rank scores; RFE recursive feature elimination

MRS exclusive Genes

UnigeneID	Description	Symbol	Cytoband	LocusLinkID	Acc No
Hs.78040	KDEL (Lys-Asp-Glu-Leu) endoplasmic reticulum protein retention receptor 1	KDELR1	19q13.3	10945	X55885.1;
Hs.223851	fibroblast growth factor 14	FGF14	13q34	2259	U66200.1;
Hs.129781	chromosome 21 open reading frame 5	C21orf5	21q22.2	9980	AJ001857.1;
Hs.110675	apolipoprotein E	APOE	19q13.2	348	Z70760.1;
Hs.65029	growth arrest-specific 1	GAS1	9q21.3-q22	2619	L13698.1;
Hs.449625	glutamate receptor, metabotropic 8	GRM8	7q31.3-q32.1	2918	U95025.1;
Hs.438702	KIAA1055 protein	KIAA1055	15q24.1	23102	NM_015079.2;
Hs.204039	aristaless-like homeobox 3	ALX3	1p21-p13	257	NM_006492.1;
Hs.111732	Fc fragment of IgG binding protein	FCGBP	19q13.1	8857	D84239.1;
Hs.211819	matrix metalloproteinase 23B	MMP23B	1p36.3	8510	AF086353.1;
Hs.370636	solute carrier family 4, sodium bicarbonate cotransporter, member 8	SLC4A8	12q13	9498	AF107099.1;
Hs.423573	discoidin domain receptor family, member 1	DDR1	6p21.3	780	X74979.1;
Hs.270143	elastin microfibril interfacer 2	EMILIN2	18p11.3	84034	AK090519.1;
Hs.288433	neurotrimin	HNT	11q25	50863	NM_016522.2;
Hs.113287	discs, large (Drosophila) homolog-associated protein 2	DLGAP2	8p23	9228	AB000275.1;
Hs.32042	aspartoacylase (aminoacylase 2, Canavan disease)	ASPA	17pter-p13	443	S67156.1;
Hs.7426	KIAA0841	KIAA0841	19q13.13	23354	BC013947.2;
Hs.458482	calcium binding protein 1 (calbrain)	CABP1	12q24.31	9478	BC015006.1;
Hs.380133	zinc fingers and homeoboxes 3	ZHX3	20q12	23051	AB007855.2;
Hs.159066	chromosome 10 open reading frame 118	C10orf118	10q26.11	55088	AF273054.1;
Hs.1424	flavin containing monooxygenase 1	FMO1	1q23-q25	2326	AK097039.1;
Hs.94234	frizzled homolog 1 (Drosophila)	FZD1	7q21	8321	AL049994.1;
Hs.416393	hypothetical protein FLJ11259	FLJ11259	12q23.3	55332	BC018435.1;
Hs.306456	PRP18 pre-mRNA processing factor 18 homolog (yeast)	PRPF18	10p14	8559	U51990.1;
Hs.103126	human T-cell leukemia virus enhancer factor	HTLF	2p22-p16	3344	U57029.1;
Hs.49658	KIAA0495	KIAA0495	1p36.32	57212	BC013872.2;
Hs.43728	glutathione peroxidase 7	GPX7	1p32	2882	AF320068.1;
Hs.267871	ATPase, H+ transporting, lysosomal V0 subunit a isoform 1	ATP6V0A1	17q21	535	U18920.1;
Hs.77813	sphingomyelin phosphodiesterase 1, acid lysosomal (acid sphingomyelinase)	SMPD1	11p15.4-p15.1	6609	BC041164.1;
Hs.158308	serine (or cysteine) proteinase inhibitor, clade I (neuroserpin), member 2	SERPINI2	3q26.1-q26.2	5276	BC027859.2;
Hs.105794	UDP-glucose ceramide glucosyltransferase-like 1	UGCGL1	2q21.1	56886	BC041098.1;
Hs.1376	hydroxysteroid (11-beta) dehydrogenase 2	HSD11B2	16q22	3291	BC036780.1;
Hs.235195	mediator subunit 25	MED25	17p11.2	55090	BC000647.2;
Hs.199292	N-acetylated alpha-linked acidic dipeptidase 2	NAALAD2	11q14.3-q21	10003	BC038840.1;
Hs.8022	TU3A protein	TU3A	3p21.1	11170	AF035283.1;
Hs.528633	mucin 4, tracheobronchial	MUC4	3q29	4585	AF058803.1;
Hs.512620	carbonic anhydrase XII	CA12	15q22	771	BC001012.2;
Hs.300772	tropomyosin 2 (beta)	TPM2	9p13.2-p13.1	7169	X06825.1;
Hs.155119	EH-domain containing 1	EHD1	11q13	10938	AF001434.1;
Hs.8867	cysteine-rich, angiogenic inducer, 61	CYR61	1p31-p22	3491	Z98053.1;
Hs.349082	Transcribed sequence with moderate similarity to protein ref:NP_008996.1 (H.sapiens) CDC37 homolog; CDC37				
Hs.427055	nuclear receptor subfamily 0, group B, member 2	NR0B2	1p36.1	8431	BC030207.1;
Hs.168762	unc-51-like kinase 2 (C. elegans)	ULK2	17p11.2	9706	BC034988.1;
Hs.209205	protein-O-mannosyltransferase 1	POMT1	9q34.1	10585	AK074874.1;
Hs.194385	signal-transducing adaptor protein-2	STAP2	19p13.3	55620	BC000795.1;
Hs.12440	Clone 24734 mRNA sequence				AF070625.1;
Hs.75353	peptidase (mitochondrial processing) alpha	PMPCA	9q34.3	23203	D21064.1;
Hs.151250	intercellular adhesion molecule 5, telencephalin	ICAM5	19p13.2	7087	U72671.1;
Hs.439911	telomerase reverse transcriptase	TERT	5p15.33	7015	AF018167.1;
Hs.120247	nuclear fragile X mental retardation protein interacting protein 1	NUFIP1	13q14	26747	BC001436.1;
Hs.272848	hypothetical protein FLJ21019	FLJ21019	17q21.31	79990	AK096767.1;
Hs.169681	death effector domain containing	DEDD	1q23.1	9191	BC016724.1;
Hs.226581	COX15 homolog, cytochrome c oxidase assembly protein (yeast)	COX15	10q24	1355	NM_078470.2;
Hs.13880	CGI-143 protein	CGI-143	1p36.13-q31.3	51027	BC002771.2;
Hs.36761	HRAS-like suppressor	HRASLS	3q29	57110	AB030816.1;
Hs.154332	ER degradation enhancer, mannosidase alpha-like 1	EDEM1	3p26.1	9695	D86967.1;
Hs.144683	CDNA clone MGC:87642 IMAGE:5265802, complete cds				BC068278.1;
Hs.433291	ARD1 homolog, N-acetyltransferase (S. cerevisiae)	ARD1	Xq28	8260	X77588.1;
Hs.93029	sparc/osteonectin, cwcv and kazal-like domains proteoglycan (testican)	SPOCK	5q31	6695	AK094489.1;
Hs.100431	chemokine (C-X-C motif) ligand 13 (B-cell chemoattractant)	CXCL13	4q21	10563	AF044197.1;
Hs.226434	beta-transducin repeat containing	BTRC	10q24.32	8945	Y14153.1;
Hs.174944	CD38 antigen (p45)	CD38	4p15	952	D84277.1;
Hs.821	biglycan	BGN	Xq28	633	U11686.1;
Hs.279943	LIM and cysteine-rich domains 1	LMCD1	3p26-p24	29995	AK074448.1;
Hs.23671	lectin, galactoside-binding, soluble, 13 (galectin 13)	LGALS13	19q13.1	29124	AY055826.1;
Hs.26176	transmembrane protein 16A	TMEM16A	11q13.2	55107	BC033036.2;
Hs.298469	angiotensin I converting enzyme (peptidyl-dipeptidase A) 1	ACE	17q23	1636	M29981.1;
Hs.20084	retinoid X receptor, alpha	RXRA	9q34.3	6256	X52773.1;
Hs.159472	v-abl Abelson murine leukemia viral oncogene homolog 2 (arg, Abelson-related	ABL2	1q24-q25	27	M35296.1;
Hs.316977	importin 8	IPO8	12p11.22	10526	U77494.1;
Hs.380460	islet cell autoantigen 1, 69kDa	ICA1	7p22	3382	U38260.1;
Hs.22393	density-regulated protein	DENR	12q24.31	8562	AF038554.1;
Hs.36	lymphotoxin alpha (TNF superfamily, member 1)	LTA	6p21.3	4049	X01393.1;
Hs.154353	solute carrier family 9 (sodium/hydrogen exchanger), isoform 7	SLC9A7	Xp11.3-p11.23	84679	AK056599.1;
Hs.153648	protein tyrosine phosphatase, receptor type, f polypeptide (PTPRF), interacting	PPFIA4	1q32.1	8497	AF034801.1;
Hs.44899	nebulin-related anchoring protein	NRAP	10q24-q26	4892	AK056969.1;
Hs.306251	v-erb-b2 erythroblastic leukemia viral oncogene homolog 3 (avian)	ERBB3	12q13	2065	M29366.1;

Hs.106346	nucleolar protein 7, 27kDa	NOL7	6p23	51406	BC023517.2;
Hs.129142	DNase II-like acid DNase	DLAD	1p22.3	58511	AF333389.1;
Hs.294101	pre-B-cell leukemia transcription factor 3	PBX3	9q33-q34	5090	X59841.1;
Hs.194714	synaptosomal-associated protein, 29kDa	SNAP29	22q11.21	9342	BC009715.1;
Hs.24545	zinc finger protein 444	ZNF444	19q13.43	55311	AF074985.1;
Hs.898	dystrophia myotonica-protein kinase	DMPK	19q13.3	1760	M87313.1;
Hs.107600	neurofilament, light polypeptide 68kDa	NEFL	8p21	4747	AY156690.1;
Hs.361041	ankyrin repeat domain 26	ANKRD26	10pter-q22.1	22852	AK001137.1;
Hs.198278	6-phosphofructo-2-kinase/fructose-2,6-biphosphatase 4	PFKFB4	3p21-p22	5210	D49818.1;
Hs.333150	zinc finger protein 286	ZNF286	17p11.2	57335	AF086305.1;
Hs.489847	Ca2+-dependent activator protein for secretion 2	CADPS2		93664	AF401638.1;
Hs.3532	nemo like kinase	NLK	17q11.2	51701	AK024686.1;
Hs.69747	fucosyltransferase 1 (galactoside 2-alpha-L-fucosyltransferase)	FUT1	19q13.3	2523	AF117899.1;
Hs.247734	protocadherin alpha 2	PCDHA2	5q31	56146	AB002343.1;
Hs.401414	Wiskott-Aldrich syndrome protein interacting protein	WASPIP	2q31.2	7456	AF031588.1;
Hs.446415	sodium channel, nonvoltage-gated 1 alpha	SCNN1A	12p13	6337	X76180.1;
Hs.375439	neuro-oncological ventral antigen 2	NOVA2	19q13.3	4858	U70477.1;
Hs.150956	exostoses (multiple)-like 1	EXTL1	1p36.1	2134	U67191.1;
Hs.104134	homeo box (H6 family) 1	HMX1	4p16.1	3166	M99587.1;
Hs.1259	asialoglycoprotein receptor 2	ASGR2	17p	433	U97197.1;
Hs.225129	N-deacetylase/N-sulfotransferase (heparan glucosaminyl) 2	NDST2	10q22	8509	BC035711.1;
Hs.238679	Ras-related GTP binding D	RRAGD	6q15-q16	58528	AJ420486.1;
Hs.95008	KiSS-1 metastasis-suppressor	KISS1	1q32	3814	AY117143.1;
Hs.111373	KIAA0423	KIAA0423	14q21.3	23116	AB007883.2;
Hs.379039	tumor suppressor deleted in oral cancer-related 1	DOC-1R	11q13	10263	AK001498.1;
Hs.105791	CDNA clone IMAGE:6186815, partial cds				BC064139.1;
Hs.193852	ATP-binding cassette, sub-family C (CFTR/MRP), member 2	ABCC2	10q24	1244	U63970.1;
Hs.408515	neuregulin 2	NRG2	5q23-q33	9542	AB005060.1;
Hs.164526	WW domain binding protein 11	WBP11	12p13.1	51729	BC023532.2;
Hs.435074	potassium voltage-gated channel, shaker-related subfamily, beta member 3	KCNAB3	17p13.1	9196	AF016411.1;
Hs.307915	ATP-binding cassette, sub-family C (CFTR/MRP), member 4	ABCC4	13q32	10257	AY133678.1;
Hs.116128	WAP four-disulfide core domain 8	WFDC8	20q13.12	90199	AF492015.1;
Hs.123036	ectonucleoside triphosphate diphosphohydrolase 2	ENTPD2	9q34	954	U91510.1;
Hs.420830	hypoxia inducible factor 3, alpha subunit	HIF3A	19q13.33	64344	NM_152794.1;
Hs.147697	nidogen 2 (osteonidogen)	NID2	14q21-q22	22795	AJ223500.1;
Hs.386791	phosphodiesterase 3A, cGMP-inhibited	PDE3A	12p12	5139	AK095203.1;
Hs.123530	actin-like 7A	ACTL7A	9q31	10881	BC014610.2;
Hs.415117	sialyltransferase 9 (CMP-NeuAc:lactosylceramide alpha-2,3-sialyltransferase; G	SIAT9	2p11.2	8869	AF344831.1;
Hs.124940	Rho family GTPase 1	RND1	12q12-q13	27289	Y07923.1;
Hs.512814	topoisomerase (DNA) III beta	TOP3B	22q11.22	8940	AL833505.1;
Hs.49344	mucolipin 3	MCOLN3	1p22.3	55283	AK093948.1;
Hs.82664	ETAA16 protein	ETAA16	2p13-p15	54465	AK026300.1;
Hs.81073	fetuin B	FETUB	3q27	26998	NM_014375.1;
Hs.152213	wingless-type MMTV integration site family, member 5A	WNT5A	3p21-p14	7474	AK090582.1;
Hs.388613	neogenin homolog 1 (chicken)	NEO1	15q22.3-q23	4756	U72391.1;
Hs.315	mucin 2, intestinal/tracheal	MUC2	11p15.5	4583	M22405.1;
Hs.446554	RAD51 homolog (RecA homolog, E. coli) (S. cerevisiae)	RAD51	15q15.1	5888	D14134.1;
Hs.159396	peptidylglycine alpha-amidating monooxygenase COOH-terminal interactor	PAMCI	12q21.32	9182	BC031589.1;
Hs.432329	hypothetical protein DKFZp564A176	DKFZp564A176	3q21.3	84202	BC017323.2;
Hs.66713	hepatitis delta antigen-interacting protein A	DIPA	11q12.1	11007	U63825.1;
Hs.444989	Transcribed sequences				
Hs.438864	natriuretic peptide receptor A/guanylate cyclase A (atrionatriuretic peptide recep	NPR1	1q21-q22	4881	S72628.1;
Hs.65424	tetranectin (plasminogen binding protein)	TNA	3p22-p21.3	7123	X64559.1;
Hs.155017	nuclear receptor interacting protein 1	NRIP1	21q11.2	8204	BC040361.1;
Hs.173374	discoidin, CUB and LCCL domain containing 2	DCBLD2	3q12.2	131566	BC016815.1;
Hs.446303	nitric oxide synthase 3 (endothelial cell)	NOS3	7q36	4846	S80791.1;
Hs.21413	solute carrier family 12, (potassium-chloride transporter) member 5	SLC12A5	20q13.12	57468	U79245.1;
Hs.222874	histone deacetylase 4	HDAC4	2q37.2	9759	AB006626.2;
Hs.349095	MRNA; cDNA DKFZp686O13119 (from clone DKFZp686O13119)				BX648277.1;
Hs.98912	chromosome 6 open reading frame 103	C6orf103	6q24.2	79747	AL832192.1;
Hs.7946	mitochondrial tumor suppressor 1	MTUS1	8p22	57509	AF086371.1;
Hs.370530	tripartite motif-containing 14	TRIM14	9q31.1	9830	AK097480.1;
Hs.24808	hypothetical protein FLJ21168	FLJ21168	1p13.1	80143	BC005934.1;
Hs.315366	protein kinase C, eta	PRKCH	14q22-q23	5583	BC037268.1;
Hs.435609	tyrosine hydroxylase	TH	11p15.5	7054	AY144494.1;
Hs.161008	karyopherin alpha 1 (importin alpha 5)	KPNA1	3q21	3836	AF035311.1;
Hs.50640	suppressor of cytokine signaling 1	SOCS1	16p13.13	8651	AB000734.1;
Hs.2288	visinin-like 1	VSNL1	2p24.3	7447	BC022012.2;
Hs.123116	solute carrier family 12 (sodium/potassium/chloride transporters), member 1	SLC12A1	15q15-q21.1	6557	BC040138.2;
Hs.88778	carbonyl reductase 1	CBR1	21q22.13	873	BC015640.2;
Hs.202300	olfactory receptor, family 51, subfamily E, member 2	OR51E2	11p15	81285	AY033942.1;
Hs.310922	BCL2-like 13 (apoptosis facilitator)	BCL2L13	22q11	23786	AF539453.1;
Hs.309257	dynein 2 light intermediate chain	D2LIC	2p25.1-p24.1	51626	BC040558.1;
Hs.438862	EPS8-like 1	EPS8L1	19q13.42	54869	AK075098.1;
Hs.352341	stress 70 protein chaperone, microsome-associated, 60kDa	STCH	21q11.1	6782	BC036370.2;
Hs.164267	dual-specificity tyrosine-(Y)-phosphorylation regulated kinase 3	DYRK3	1q32	8444	Y12735.2;
Hs.79241	B-cell CLL/lymphoma 2	BCL2	18q21.33	596	M14745.1;
Hs.252839	interferon-induced protein with tetratricopeptide repeats 5	IFIT5	10q23.32	24138	BC025786.1;
Hs.153003	serine/threonine kinase 16	STK16	2q34-q37	8576	AK096954.1;
Hs.159201	thymosin, beta 4, Y-linked	TMSB4Y	Yq11.221	9087	AF000989.1;

Hs.377070	glutamate receptor, ionotrophic, AMPA 3	GRIA3	Xq25-q26	2892	U10302.1;
Hs.11494	fibulin 5	FBLN5	14q32.1	10516	AL110148.1;
Hs.276808	mannosyl (beta-1,4-)-glycoprotein beta-1,4-N-acetylglucosaminyltransferase	MGAT3	22q13.1	4248	L48489.1;
Hs.26630	ATP-binding cassette, sub-family A (ABC1), member 3	ABCA3	16p13.3	21	U78735.1;
Hs.436928	deoxyribonuclease I	DNASE1	16p13.3	1773	D17270.1;
Hs.76253	spinocerebellar ataxia 2 (olivopontocerebellar ataxia 2, autosomal dominant, at	SCA2	12q24.1	6311	Y08262.1;
Hs.222707	KIAA1718 protein	KIAA1718	7q33-q35	80853	AK057208.1;
Hs.119591	adaptor-related protein complex 2, sigma 1 subunit	AP2S1	19q13.2-q13.3	1175	X97074.1;
Hs.85482	ubiquitin specific protease 13 (isopeptidase T-3)	USP13	3q26.2-q26.3	8975	U75362.1;
Hs.118057	placenta-specific 4	PLAC4	21q22.3	191585	L13197.1;
Hs.59260	solute carrier family 6 (neurotransmitter transporter), member 16	SLC6A16	19q13.1-q13.4	28968	BC034948.1;
Hs.376810	zinc finger protein 23 (KOX 16)	ZNF23	16q22	7571	NM_145911.1;
Hs.405467	DNA glycosylase hFPG2	FLJ10858	4q34.3	55247	AB079071.1;
Hs.9622	mitochondrial ribosomal protein S18A	MRPS18A	6p21.3	55168	BC010364.1;
Hs.321214	hypothetical protein DKFZp564D0478	DKFZP564D0478	1p35.3	84065	AK095889.1;
Hs.73875	fumarylacetoacetate hydrolase (fumarylacetoacetase)	FAH	15q23-q25	2184	S63548.1;

Table B: The list of genes found exclusively in the MRS integrated set.

This list contains only those genes found in an RFE analysis of both leukemia data sets integrated by MRS, that were not found in both single set analyses.
Abbreviations: MRS, median rank scores; RFE recursive feature elimination

MRS Intersect Bullinger and Valk, but not in combined set

UnigeneID	Description	Symbol	Cytoband	LocusLinkID	Acc No
Hs.149609	integrin, alpha 5 (fibronectin receptor, alpha polypeptide)	ITGA5	12q11-q13	3678	X06256.1;
Hs.2340	junction plakoglobin	JUP	17q21	3728	M23410.1;
Hs.3416	adipose differentiation-related protein	ADFP	9p22.1-p21.3	123	X97324.1;
Hs.2463	angiopoietin 1	ANGPT1	8q22.3-q23	284	U83508.1;
Hs.351316	transmembrane 4 superfamily member 1	TM4SF1	3q21-q25	4071	X75684.1;
Hs.96731	huntingtin interacting protein-1-related	HIP1R	12q24	9026	BC011354.2;
Hs.82273	hypothetical protein FLJ20152	FLJ20152	5p15.1	54463	AL832438.1;
Hs.190783	histidine ammonia-lyase	HAL	12q22-q24.1	3034	D16626.1;
Hs.166011	catenin (cadherin-associated protein), delta 1	CTNND1	11q11	1500	Z36799.1;
Hs.511765	GLI pathogenesis-related 1 (glioma)	GLIPR1	12q21.1	11010	U16307.3;
Hs.12	carcinoembryonic antigen-related cell adhesion molecule 4	CEACAM4	19q13.2	1089	D90276.1;
Hs.350927	solute carrier family 25 (mitochondrial carrier; adenine nucleotide translocator), member 6	SLC25A6	Xp22.32	293	AF076617.1;
Hs.76873	hyaluronoglucosaminidase 2	HYAL2	3p21.3	8692	AK092449.1;
Hs.528644	hypothetical protein FLJ11127	FLJ11127	5p15.2	54491	BC011524.1;
Hs.69293	hexosaminidase B (beta polypeptide)	HEXB	5q13	3074	M19735.1;
Hs.48778	chromosome 1 open reading frame 24	C1orf24	1q25	116496	AK095547.1;
Hs.44313	v-rel reticuloendotheliosis viral oncogene homolog (avian)	REL	2p13-p12	5966	X75042.1;
Hs.352662	golgi phosphoprotein 2	GOLPH2	9q21.33	51280	AK075542.1;
Hs.76640	response gene to complement 32	RGC32	13q13.3	28984	AK091099.1;
Hs.436944	sprouty homolog 1, antagonist of FGF signaling (Drosophila)	SPRY1	4q28.1	10252	AF041037.1;

Table C: The list of genes found by RFE in both single-set leukemia analyses, but not in analysis of both sets integrated by MRS.

This list is the intersection of the three gene lists generated on the data of Bullinger et al, Valk et al without the genes also found in the list generated on the data of both studies integrated by MRS. Abbreviations: MRS, median rank scores; RFE recursive feature elimination

QD intersect of all sets

UnigeneID	Description	Symbol	Cytoband	LocusLinkID	Acc No
Hs.26985	disrupted in schizophrenia 1	DISC1	1q42.1	27185	AJ506177.1;
Hs.1145	Wilms tumor 1	WT1	11p13	7490	X51630.1;
Hs.76536	transducin (beta)-like 1X-linked	TBL1X	Xp22.3	6907	Y12781.1;
Hs.412117	annexin A6	ANXA6	5q32-q34	309	J03578.1;
Hs.173380	CK2 interacting protein 1; HQ0024c protein	CKIP-1	1q21.3	51177	BC023533.2;
Hs.50216	zinc finger protein (ZFD25)	ZFD25	7q11.2	51427	AB014735.1;
Hs.211588	POU domain, class 4, transcription factor 1	POU4F1	13q21.1-q22	5457	BC041859.1;
Hs.3407	protein kinase (cAMP-dependent, catalytic) inhibitor gamma	PKIG	20q12-q13.1	11142	AF182032.1;
Hs.278429	chromosome 9 open reading frame 78	C9orf78	9q34.2	51759	AF007871.1;
Hs.370374	YY1 associated protein	YAP	1q22	55249	AF203474.1;
Hs.151449	suppression of tumorigenicity 18 (breast carcinoma) (zinc finger protein)	ST18	8q11.22	9705	AB011107.1;
Hs.8309	likely ortholog of mouse membrane bound C2 domain containing protein	MBC2	12q13.13	23344	BC013880.2;
Hs.15871	lysophosphatidic acid phosphatase	ACP6	1q21	51205	BC034686.1;
Hs.75485	ornithine aminotransferase (gyrate atrophy)	OAT	10q26	4942	BC016928.1;
Hs.421474	ATP-binding cassette, sub-family A (ABC1), member 5	ABCA5	17q24.3	23461	NM_172232.1;
Hs.14158	copine III	CPNE3	8q21.2	8895	BC036242.1;
Hs.154437	phosphodiesterase 2A, cGMP-stimulated	PDE2A	11q13.3	5138	U67733.1;
Hs.16134	serine/threonine kinase 10	STK10	5q35.1	6793	AK092814.1;
Hs.129959	interleukin 17 receptor C	IL17RC	3p25.3	84818	NM_153460.1;
Hs.153563	lymphocyte antigen 75	LY75	2q24	4065	NM_002349.1;
Hs.371698	RAE1 RNA export 1 homolog (S. pombe)	RAE1	20q13.31	8480	U84720.1;
Hs.226117	H1 histone family, member 0	H1F0	22q13.1	3005	AK091372.1;
Hs.416848	cathepsin W (lymphopain)	CTSW	11q13.1	1521	AF013611.1;
Hs.368746	neuropilin 2	NRP2	2q33.3	8828	AF016098.1;

Table D: The list of genes found by RFE in all QD sets.
This list is the intersection of the three gene lists generated on the data of Bullinger et al, Valk et al and of the list generated on the data of both studies integrated by QD. Abbreviations: QD, quantile discretization; RFE recursive feature elimination

QD exclusive genes

UnigeneID	Description	Symbol	Cytoband	LocusLinkID	Acc No
Hs.407934	neuron navigator 2	NAV2	11p15.1	89797	BC035903.1;
Hs.223851	fibroblast growth factor 14	FGF14	13q34	2259	U66200.1;
Hs.287460	PDZ domain containing 3	PDZK3	5p13.3	23037	AB002298.2;
Hs.129781	chromosome 21 open reading frame 5	C21orf5	21q22.2	9980	AJ001857.1;
Hs.7984	pleckstrin homology, Sec7 and coiled-coil domains 3	PSCD3	7p22.2	9265	AJ223957.1;
Hs.178499	HSPC063 protein	HSPC063	11q24.3	29068	BC040658.1;
Hs.65029	growth arrest-specific 1	GAS1	9q21.3-q22	2619	L13698.1;
Hs.438702	KIAA1055 protein	KIAA1055	15q24.1	23102	NM_015079.2;
Hs.270143	elastin microfibril interfacer 2	EMILIN2	18p11.3	84034	AK090519.1;
Hs.70669	HMP19 protein	HMP19	5q35.2	51617	AK098398.1;
Hs.32042	aspartoacylase (aminoacylase 2, Canavan disease)	ASPA	17pter-p13	443	S67156.1;
Hs.458482	calcium binding protein 1 (calbrain)	CABP1	12q24.31	9478	BC015006.1;
Hs.380133	zinc fingers and homeoboxes 3	ZHX3	20q12	23051	AB007855.2;
Hs.37558	riboflavin kinase	RFK	9q21.31	55312	AF038172.1;
Hs.370480	ATP-binding cassette, sub-family B (MDR/TAP), member 7	ABCB7	Xq12-q13	22	BC006323.2;
Hs.103126	human T-cell leukemia virus enhancer factor	HTLF	2p22-p16	3344	U57029.1;
Hs.158530	Rap guanine nucleotide exchange factor (GEF)-like 1	RAPGEFL1	17q21.2	51195	U78518.1;
Hs.49658	KIAA0495	KIAA0495	1p36.32	57212	BC013872.2;
Hs.77813	sphingomyelin phosphodiesterase 1, acid lysosomal (acid sphingomyelinase)	SMPD1	11p15.4-p15.1	6609	BC041164.1;
Hs.158308	serine (or cysteine) proteinase inhibitor, clade I (neuroserpin), member 2	SERPINI2	3q26.1-q26.2	5276	BC027859.2;
Hs.117874	paired basic amino acid cleaving system 4	PACE4	15q26	5046	D87995.1;
Hs.1376	hydroxysteroid (11-beta) dehydrogenase 2	HSD11B2	16q22	3291	BC036780.1;
Hs.1395	early growth response 2 (Krox-20 homolog, Drosophila)	EGR2	10q21.1	1959	BC035625.1;
Hs.31653	Rho-related BTB domain containing 3	RHOBTB3	5q15	22836	BC041337.2;
Hs.512681	sodium channel, nonvoltage-gated 1, delta	SCNN1D	1p36.3-p36.2	6339	U38254.1;
Hs.235195	mediator subunit 25	MED25	17p11.2	55090	BC000647.2;
Hs.199292	N-acetylated alpha-linked acidic dipeptidase 2	NAALAD2	11q14.3-q21	10003	BC038840.1;
Hs.48384	heparan sulfate (glucosamine) 3-O-sulfotransferase 3B1	HS3ST3B1	17p12-p11.2	9953	AL832823.1;
Hs.282938	collagen, type IV, alpha 4	COL4A4	2q35-q37	1286	X81053.1;
Hs.334475	hypothetical protein FLJ10634	FLJ10634	15q14	55192	BC000048.2;
Hs.8022	TU3A protein	TU3A	3p21.1	11170	AF035283.1;
Hs.300772	tropomyosin 2 (beta)	TPM2	9p13.2-p13.1	7169	X06825.1;
Hs.42343	MYST histone acetyltransferase 1	MYST1	16p11.2	84148	BC037773.1;
Hs.155119	EH-domain containing 1	EHD1	11q13	10938	AF001434.1;
Hs.8867	cysteine-rich, angiogenic inducer, 61	CYR61	1p31-p22	3491	Z98053.1;
Hs.82568	cytochrome P450, family 27, subfamily A, polypeptide 1	CYP27A1	2q33-qter	1593	X59812.1;
Hs.349082	Transcribed sequence with moderate similarity to protein ref:NP_008996.1 (H.sapiens) CDC37 homolog; CDC37				
Hs.278489	matrilin 4	MATN4	20q13.1-q13.2	8785	AK074495.1;
Hs.427055	nuclear receptor subfamily 0, group B, member 2	NR0B2	1p36.1	8431	BC030207.1;
Hs.168762	unc-51-like kinase 2 (C. elegans)	ULK2	17p11.2	9706	BC034988.1;
Hs.331141	aldehyde dehydrogenase 2 family (mitochondrial)	ALDH2	12q24.2	217	M54931.1;
Hs.2393	phosphorylase kinase, alpha 1 (muscle)	PHKA1	Xq12-q13	5255	X73878.1;
Hs.194385	signal-transducing adaptor protein-2	STAP2	19p13.3	55620	BC000795.1;
Hs.150206	histone 1, H1a	HIST1H1A	6p21.3	3024	BC069492.1;
Hs.2799	hyaluronan and proteoglycan link protein 1	HAPLN1	5q14.3	1404	X78076.1;
Hs.12440	Clone 24734 mRNA sequence				AF070625.1;
Hs.1281	complement component 5	C5	9q33-q34	727	M57729.1;
Hs.300863	l(3)mbt-like (Drosophila)	L3MBTL	20q13.12	26013	BC039820.1;
Hs.7122	scrapie responsive protein 1	SCRG1	4q31-q32	11341	BC017583.2;
Hs.151250	intercellular adhesion molecule 5, telencephalin	ICAM5	19p13.2	7087	U72671.1;
Hs.439911	telomerase reverse transcriptase	TERT	5p15.33	7015	AF018167.1;
Hs.272848	hypothetical protein FLJ21019	FLJ21019	17q21.31	79990	AK096767.1;
Hs.226581	COX15 homolog, cytochrome c oxidase assembly protein (yeast)	COX15	10q24	1355	NM_078470.2;
Hs.13880	CGI-143 protein	CGI-143	1p36.13-q31.3	51027	BC002771.2;
Hs.53250	bone morphogenetic protein receptor, type II (serine/threonine kinase)	BMPR2	2q33-q34	659	U25110.1;
Hs.85567	suppressor of variegation 3-9 homolog 2 (Drosophila)	SUV39H2	10p13	79723	AL834488.1;
Hs.10082	potassium intermediate/small conductance calcium-activated channel, subfamily N, member 4	KCNN4	19q13.2	3783	AF022797.1;
Hs.226434	beta-transducin repeat containing	BTRC	10q24.32	8945	Y14153.1;
Hs.193143	KIAA1069 protein	KIAA1069	3q25.2-q25.31	23007	AK093601.1;
Hs.174944	CD38 antigen (p45)	CD38	4p15	952	D84277.1;
Hs.12040	STE20-like kinase	JIK	12q	51347	AF328731.1;
Hs.25040	zinc finger protein 239	ZNF239	10q11.22-q11.23	8187	X82128.1;
Hs.279943	LIM and cysteine-rich domains 1	LMCD1	3p26-p24	29995	AK074448.1;
Hs.414110	potassium voltage-gated channel, Shal-related subfamily, member 3	KCND3	1p13.3	3752	AF048713.1;
Hs.98314	MRNA; cDNA DKFZp586L0120 (from clone DKFZp586L0120)				AK074200.1;
Hs.434406	zinc finger protein 539	ZNF539	19p12	399655	AL832713.1;
Hs.149376	retinoschisis (X-linked, juvenile) 1	RS1	Xp22.2-p22.1	6247	AF014459.1;
Hs.298469	angiotensin I converting enzyme (peptidyl-dipeptidase A) 1	ACE	17q23	1636	M29981.1;
Hs.79136	solute carrier family 39 (zinc transporter), member 6	SLC39A6	18q12.2	25800	BC039498.1;
Hs.406074	PRKC, apoptosis, WT1, regulator	PAWR	12q21	5074	AK024560.1;
Hs.159472	v-abl Abelson murine leukemia viral oncogene homolog 2 (arg, Abelson-related gene)	ABL2	1q24-q25	27	M35296.1;
Hs.316977	importin 8	IPO8	12p11.22	10526	U77494.1;
Hs.380460	islet cell autoantigen 1, 69kDa	ICA1	7p22	3382	U38260.1;
Hs.1634	cell division cycle 25A	CDC25A	3p21	993	AY137580.1;
Hs.154353	solute carrier family 9 (sodium/hydrogen exchanger), isoform 7	SLC9A7	Xp11.3-p11.23	84679	AK056599.1;
Hs.44899	nebulin-related anchoring protein	NRAP	10q24-q26	4892	AK056969.1;
Hs.306251	v-erb-b2 erythroblastic leukemia viral oncogene homolog 3 (avian)	ERBB3	12q13	2065	M29366.1;
Hs.106346	nucleolar protein 7, 27kDa	NOL7	6p23	51406	BC023517.2;
Hs.380819	phospholipase D1, phophatidylcholine-specific	PLD1	3q26	5337	AK091897.1;
Hs.2303	erythropoietin	EPO	7q22	2056	X02157.1;
Hs.20423	CCR4-NOT transcription complex, subunit 4	CNOT4	7q22-qter	4850	BC035590.1;
Hs.294101	pre-B-cell leukemia transcription factor 3	PBX3	9q33-q34	5090	X59841.1;
Hs.446568	TRK-fused gene	TFG	3q11-q12	10342	BC023599.2;

Hs.75256	regulator of G-protein signalling 1	RGS1	1q31	5996	S59049.1;
Hs.419815	epidermal growth factor (beta-urogastrone)	EGF	4q25	1950	X04571.1;
Hs.333150	zinc finger protein 286	ZNF286	17p11.2	57335	AF086305.1;
Hs.489847	Ca2+-dependent activator protein for secretion 2	CADPS2		93664	AF401638.1;
Hs.928	proteinase 3 (serine proteinase, neutrophil, Wegener granulomatosis autoantigen)	PRTN3	19p13.3	5657	X56606.1;
Hs.3532	nemo like kinase	NLK	17q11.2	51701	AK024686.1;
Hs.199263	serine threonine kinase 39 (STE20/SPS1 homolog, yeast)	STK39	2q31.1	27347	AF017635.1;
Hs.31095	microtubule-associated protein 4	MAP4	3p21	4134	U19727.1;
Hs.247734	protocadherin alpha 2	PCDHA2	5q31	56146	AB002343.1;
Hs.375439	neuro-oncological ventral antigen 2	NOVA2	19q13.3	4858	U70477.1;
Hs.150956	exostoses (multiple)-like 1	EXTL1	1p36.1	2134	U67191.1;
Hs.425777	ubiquitin-conjugating enzyme E2L 6	UBE2L6	11q12	9246	AF031141.1;
Hs.225129	N-deacetylase/N-sulfotransferase (heparan glucosaminyl) 2	NDST2	10q22	8509	BC035711.1;
Hs.95008	KiSS-1 metastasis-suppressor	KISS1	1q32	3814	AY117143.1;
Hs.193852	ATP-binding cassette, sub-family C (CFTR/MRP), member 2	ABCC2	10q24	1244	U63970.1;
Hs.311097	exportin 4	XPO4	13q11	64328	AL831819.2;
Hs.81118	leukotriene A4 hydrolase	LTA4H	12q22	4048	BC032528.1;
Hs.243987	GATA binding protein 4	GATA4	8p23.1-p22	2626	D78260.1;
Hs.227489	syntrophin associated serine/threonine kinase	SAST	19p13.2	22983	BC027985.1;
Hs.436852	fibroblast activation protein, alpha	FAP	2q23	2191	U76833.1;
Hs.184916	clathrin, heavy polypeptide-like 1	CLTCL1	22q11.2	8218	L77568.1;
Hs.758	RAS p21 protein activator (GTPase activating protein) 1	RASA1	5q13.3	5921	BC033015.2;
Hs.123036	ectonucleoside triphosphate diphosphohydrolase 2	ENTPD2	9q34	954	U91510.1;
Hs.420830	hypoxia inducible factor 3, alpha subunit	HIF3A	19q13.33	64344	NM_152794.1;
Hs.386791	phosphodiesterase 3A, cGMP-inhibited	PDE3A	12p12	5139	AK095203.1;
Hs.123530	actin-like 7A	ACTL7A	9q31	10881	BC014610.2;
Hs.415117	sialyltransferase 9 (CMP-NeuAc:lactosylceramide alpha-2,3-sialyltransferase; GM3 synthase)	SIAT9	2p11.2	8869	AF344831.1;
Hs.410113	restin (Reed-Steinberg cell-expressed intermediate filament-associated protein)	RSN	12q24.3	6249	M97501.1;
Hs.128856	scavenger receptor class A, member 3	SCARA3	8p21	51435	NM_016240.2;
Hs.49344	mucolipin 3	MCOLN3	1p22.3	55283	AK093948.1;
Hs.82664	ETAA16 protein	ETAA16	2p13-p15	54465	AK026300.1;
Hs.290873	protein phosphatase, EF hand calcium-binding domain 2	PPEF2	4q21.21	5470	AF023457.1;
Hs.444003	arylacetamide deacetylase (esterase)	AADAC	3q21.3-q25.2	13	L32179.1;
Hs.388613	neogenin homolog 1 (chicken)	NEO1	15q22.3-q23	4756	U72391.1;
Hs.1166	thrombopoietin (myeloproliferative leukemia virus oncogene ligand, megakaryocyte growth and	THPO	3q27	7066	D32047.1;
Hs.315	mucin 2, intestinal/tracheal	MUC2	11p15.5	4583	M22405.1;
Hs.317335	ClpP caseinolytic protease, ATP-dependent, proteolytic subunit homolog (E. coli)	CLPP	19p13.3	8192	BC002956.1;
Hs.444989	Transcribed sequences				
Hs.1939	v-erb-a erythroblastic leukemia viral oncogene homolog 4 (avian)	ERBB4	2q33.3-q34	2066	L07868.1;
Hs.282326	Down syndrome critical region gene 1	DSCR1	21q22.1-q22.2	1827	U85267.2;
Hs.26550	retinoid X receptor, gamma	RXRG	1q22-q23	6258	BC012063.1;
Hs.435639	ADP-ribosylation factor 4	ARF4	3p21.2-p21.1	378	AF493883.1;
Hs.155017	nuclear receptor interacting protein 1	NRIP1	21q11.2	8204	BC040361.1;
Hs.351798	FLJ20298 protein	FLJ20298	Xq22.3	54885	AK091248.1;
Hs.446303	nitric oxide synthase 3 (endothelial cell)	NOS3	7q36	4846	S80791.1;
Hs.101174	microtubule-associated protein tau	MAPT	17q21.1	4137	BC040444.1;
Hs.98912	chromosome 6 open reading frame 103	C6orf103	6q24.2	79747	AL832192.1;
Hs.24808	hypothetical protein FLJ21168	FLJ21168	1p13.1	80143	BC005934.1;
Hs.21572	KIAA0644 gene product	KIAA0644	7p15.1	9865	BC036337.1;
Hs.315366	protein kinase C, eta	PRKCH	14q22-q23	5583	BC037268.1;
Hs.435609	tyrosine hydroxylase	TH	11p15.5	7054	AY144494.1;
Hs.21542	ATP/GTP binding protein 1	AGTPBP1	9q22.1	23287	AJ420433.1;
Hs.161008	karyopherin alpha 1 (importin alpha 5)	KPNA1	3q21	3836	AF035311.1;
Hs.50640	suppressor of cytokine signaling 1	SOCS1	16p13.13	8651	AB000734.1;
Hs.2288	visinin-like 1	VSNL1	2p24.3	7447	BC022012.2;
Hs.135554	heat shock protein (hsp110 family)	APG-1	4q28	22824	BC040560.1;
Hs.88778	carbonyl reductase 1	CBR1	21q22.13	873	BC015640.2;
Hs.75334	exostoses (multiple) 2	EXT2	11p12-p11	2132	U72263.1;
Hs.202300	olfactory receptor, family 51, subfamily E, member 2	OR51E2	11p15	81285	AY033942.1;
Hs.66170	SET and MYND domain containing 2	SMYD2	1q32.3	56950	BC039890.1;
Hs.438862	EPS8-like 1	EPS8L1	19q13.42	54869	AK075098.1;
Hs.352341	stress 70 protein chaperone, microsome-associated, 60kDa	STCH	21q11.1	6782	BC036370.2;
Hs.79241	B-cell CLL/lymphoma 2	BCL2	18q21.33	596	M14745.1;
Hs.153003	serine/threonine kinase 16	STK16	2q34-q37	8576	AK096954.1;
Hs.238648	oncostatin M receptor	OSMR	5p13.2	9180	U60805.1;
Hs.276808	mannosyl (beta-1,4-)-glycoprotein beta-1,4-N-acetylglucosaminyltransferase	MGAT3	22q13.1	4248	L48489.1;
Hs.26630	ATP-binding cassette, sub-family A (ABC1), member 3	ABCA3	16p13.3	21	U78735.1;
Hs.184664	hypothetical protein FLJ13611	FLJ13611	5q12.3	80006	AF088056.1;
Hs.436928	deoxyribonuclease I	DNASE1	16p13.3	1773	D17270.1;
Hs.85482	ubiquitin specific protease 13 (isopeptidase T-3)	USP13	3q26.2-q26.3	8975	U75362.1;
Hs.59260	solute carrier family 6 (neurotransmitter transporter), member 16	SLC6A16	19q13.1-q13.4	28968	BC034948.1;
Hs.376810	zinc finger protein 23 (KOX 16)	ZNF23	16q22	7571	NM_145911.1;
Hs.405467	DNA glycosylase hFPG2	FLJ10858	4q34.3	55247	AB079071.1;
Hs.73875	fumarylacetoacetate hydrolase (fumarylacetoacetase)	FAH	15q23-q25	2184	S63548.1;
Hs.321214	hypothetical protein DKFZp564D0478	DKFZP564D0478	1p35.3	84065	AK095889.1;

Table E: The list of genes found exclusively in the QD integrated set.

This list contains only those genes found in an RFE analysis of both leukemia data sets integrated by QD, that were not found in both single set analyses.

Abbreviations: QD, quantile discretization; RFE recursive feature elimination

QD intersect Bullinger and Valk, but not in combined set

UnigeneID	Description	Symbol	Cytoband	LocusLinkID	Acc No
Hs.292541	zinc finger, DHHC domain containing 14	ZDHHC14	6q25.3	79683	BC040707.1;
Hs.149609	integrin, alpha 5 (fibronectin receptor, alpha polypeptide)	ITGA5	12q11-q13	3678	X06256.1;
Hs.12862	radical fringe homolog (Drosophila)	RFNG	17q25	5986	AF038196.1;
Hs.2340	junction plakoglobin	JUP	17q21	3728	M23410.1;
Hs.415067	coronin, actin binding protein, 1A	CORO1A	16p12.1	11151	AK096332.1;
Hs.2463	angiopoietin 1	ANGPT1	8q22.3-q23	284	U83508.1;
Hs.96731	huntingtin interacting protein-1-related	HIP1R	12q24	9026	BC011354.2;
Hs.76873	hyaluronoglucosaminidase 2	HYAL2	3p21.3	8692	AK092449.1;
Hs.528644	hypothetical protein FLJ11127	FLJ11127	5p15.2	54491	BC011524.1;
Hs.436348	potassium channel tetramerisation domain containing 15	KCTD15	19q13.12	79047	AK096843.1;
Hs.190783	histidine ammonia-lyase	HAL	12q22-q24.1	3034	D16626.1;
Hs.48778	chromosome 1 open reading frame 24	C1orf24	1q25	116496	AK095547.1;
Hs.99915	androgen receptor (dihydrotestosterone receptor; testicular feminization;	AR	Xq11.2-q12	367	AF321914.1;

Table F: The list of genes found by RFE in both single-set leukemia analyses, but not in analysis of both sets integrated by QD. This list is the intersection of the three gene lists generated on the data of Bullinger et al, Valk et al without the genes also found in the list generated on the data of both studies integrated by QD. Abbreviations: QD, quantile discretization, RFE, recursive feature elimination
