# Supplementary material for: Cross-platform analysis of cancer microarray data improves gene expression based classification of phenotypes
Source: BMC Bioinformatics. 2005 Nov 4;6:265. doi: 10.1186/1471-2105-6-265 (PMC1312314; doi:10.1186/1471-2105-6-265)
Supplement: Additional File 5 — Workflow for calculation of the presented class prediction accuracies. (a) Classifier performance evaluation on an independent data set as applied for calculation of the results presented in Figure 3 and Additional File 1. (b) Classifier performance evaluation by repeated cross validation as applied for calculation of the results presented in Table 2 and Additional File 2. [file 1471-2105-6-265-S5.pdf]

a

Classifier performance evaluation by independent test set

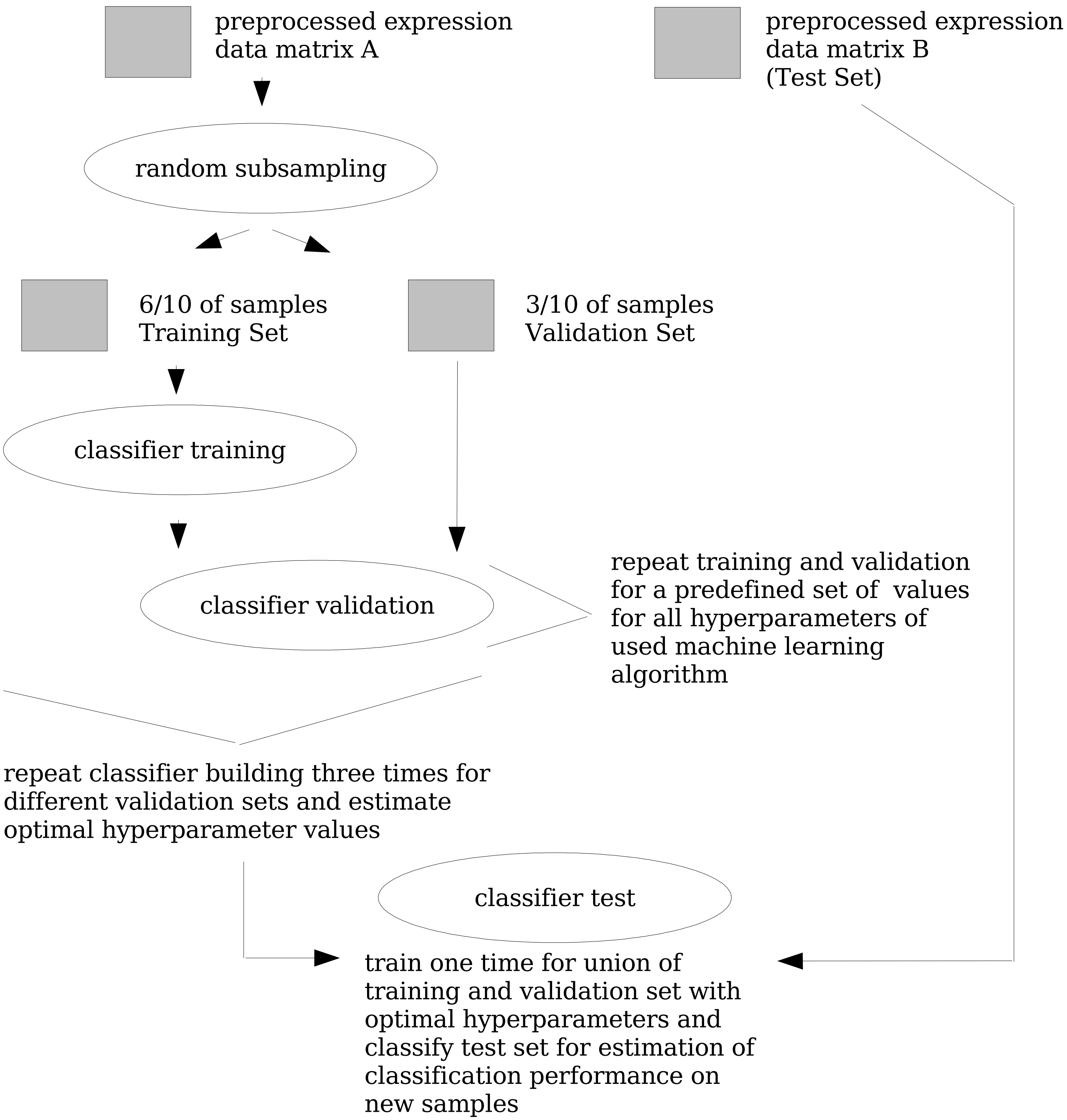

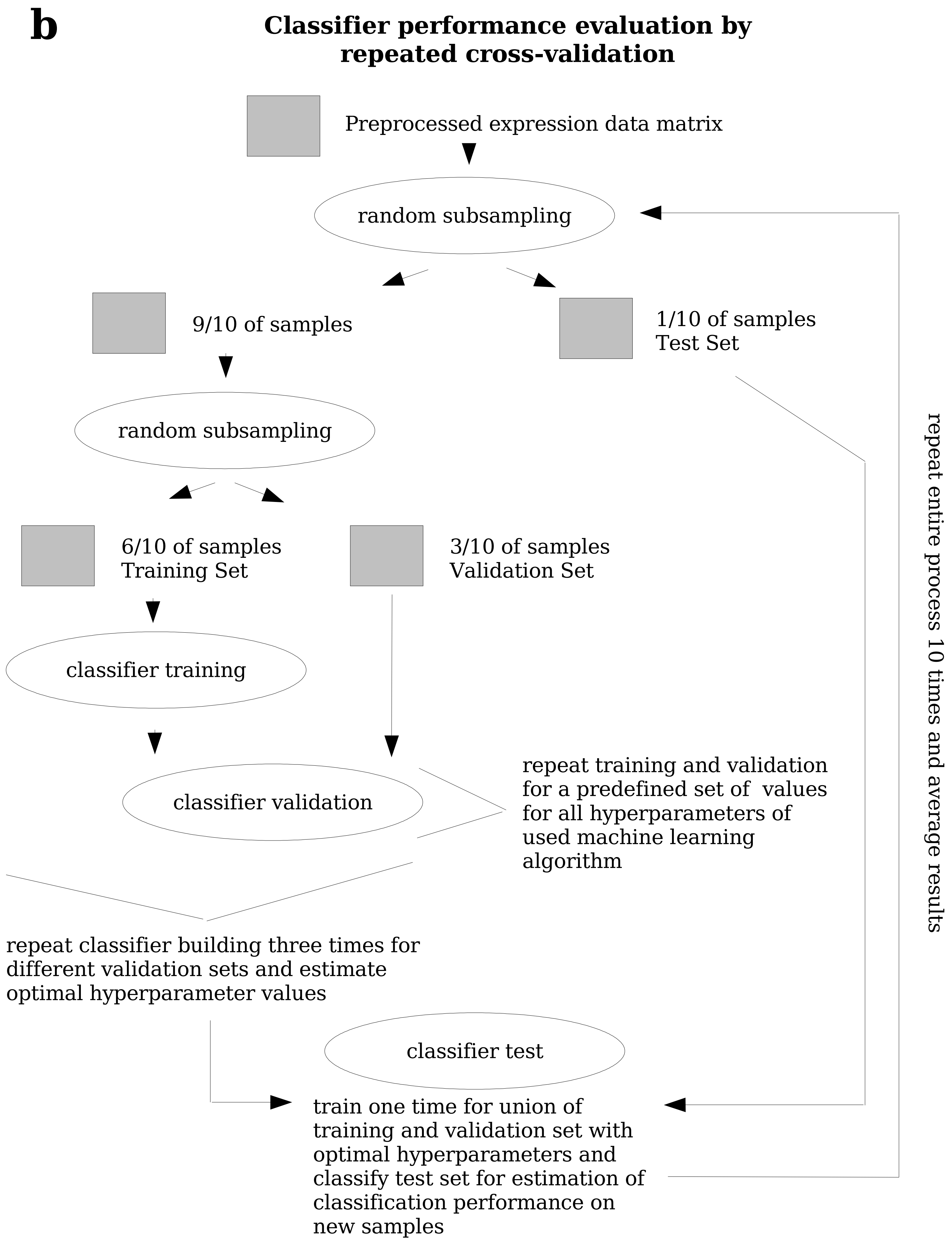
b
Classifier performance evaluation by repeated cross-validation
Preprocessed expression data matrix
random subsampling
9/10 of samples
1/10 of samples
Test Set
random subsampling
6/10 of samples
Training Set
3/10 of samples
Validation Set
classifier training
classifier validation
repeat training and validation for a predefined set of values for all hyperparameters of used machine learning algorithm
repeat classifier building three times for different validation sets and estimate optimal hyperparameter values
classifier test
train one time for union of training and validation set with optimal hyperparameters and classify test set for estimation of classification performance on new samples
repeat entire process 10 times and average results
